# Epigenetic changes and serotype-specific responses of alveolar type II epithelial cells to *Streptococcus pneumoniae* in resolving influenza A virus infection

**DOI:** 10.1186/s12964-025-02284-y

**Published:** 2025-06-12

**Authors:** Julia D Boehme, Andreas Jeron, Kristin Schultz, Lars Melcher, Katharina Schott, Elif Gelmez, Andrea Kröger, Sabine Stegemann-Koniszewski, Dunja Bruder

**Affiliations:** 1 Infection Immunology Group, Institute of Medical Microbiology and Hospital Hygiene, Health Campus Immunology, Infectiology and Inflammation, Otto-von-Guericke University, Magdeburg, Germany; 2 Immune Regulation Group, Helmholtz Centre for Infection Research, Braunschweig, Germany; 3 Infection Genetics Group, Helmholtz Centre for Infection Research, Braunschweig, Germany; 4 Molecular Microbiology Group, Institute of Medical Microbiology and Hospital Hygiene, Health Campus Immunology, Infectiology and Inflammation, Otto-von-Guericke University, Magdeburg, Germany; 5 Innate Immunity and Infection Group, Helmholtz Centre for Infection Research, Braunschweig, Germany; 6 Experimental Pneumology Group, Department of Pneumology, Health Campus Immunology, Infectiology and Inflammation, Otto-von-Guericke University, Magdeburg, Germany

**Keywords:** Influenza A virus, *Streptococcus pneumoniae*, Alveolar type II epithelial cells, Interferon response, Secondary infection, Gene co-expression network, ARACNE, ATAC sequencing, Trained innate immunity

## Abstract

**Background:**

Pneumococcal pneumonia following influenza A virus (IAV) infection is a synergistic complication with high mortality in which IAV infection modulates host antibacterial responses and affects bacterial invasiveness of *Streptococcus pneumoniae* (*S. pn.*). IAV-mediated effects can last beyond viral clearance. In acute IAV pneumonia, alveolar type II epithelial cells (AECII) are primary targets for viral replication and contribute to the immune response. Our study addresses sustained effects of IAV infection on AECII and consequences for their response towards different serotypes of *S. pn.*

**Methods:**

We analyzed bacterial loads, respiratory inflammation and AECII gene transcription profiling in mice infected with IAV and/or one of three *S. pn.* serotypes of varying invasiveness (4 > 7F > 19F). We inferred a scale-free-like ARACNE gene co-expression network on AECII transcriptional regulation under these conditions. We performed Western blotting for protein expression of interferon signaling components in AECII. We additionally performed ATAC-seq analysis of AECII isolated 14 days following IAV infection.

**Results:**

Previous IAV infection rendered the lung susceptible to invasive *S. pn.* infection with serotype 4 and the mildly invasive 7F but not 19F. Particularly secondary infection with 7F induced exacerbated inflammatory responses as compared to bacterial infection alone, marked by increased protein expression of type I and II interferons. AECII gene co-expression network revealed interferon-response network modules. Network-mapping unfolded *S. pn.* serotype-specific transcriptional network responses/usage and secondary *S. pn.* infection was found to abrogate an IAV-induced AECII proliferative configuration. Enhanced expression of several ARACNE network genes were found to be associated with increased chromatin accessibility at their promoter regions.

**Conclusions:**

Our study demonstrates AECII to retain a sustained IAV-associated configuration with epigenetic involvement, affecting their proliferation and serotype-specifically intensifying their transcriptional response, mainly to interferons, in secondary *S. pn.* infection. In a broader context, our results suggest the concepts of peripheral inflammatory imprinting and trained innate immunity to apply to cells of the respiratory epithelium in the context of subsequent viral/bacterial challenges.

**Supplementary Information:**

The online version contains supplementary material available at 10.1186/s12964-025-02284-y.

## Abstract (plain language summary)

After an influenza A virus (IAV) infection, the lungs become more vulnerable to bacterial infections caused by *Streptococcus pneumoniae* (*S. pn.*). This effect can last even after the virus is cleared. Alveolar type II epithelial cells (AECII) in the lungs are primary targets for IAV and play a crucial role in the immune response. Our study examines how IAV infection affects AECII and their response to different *S. pn.* serotypes. We studied mice infected with IAV and three *S. pn.* serotypes to analyze bacterial loads, inflammation, and gene activity in AECII. We discovered that previous IAV infection made lungs more susceptible to certain *S. pn.* serotypes. Specifically, secondary infection with serotype 7F caused a stronger inflammatory response, marked by increased type I and II interferons. Our research revealed that AECII maintain changes caused by IAV, affecting their growth and response to bacteria. These changes are linked to alterations in gene accessibility within AECII. In summary, IAV leaves lasting effects on lung cells, making them more responsive to specific bacterial infections through enhanced interferon responses. This supports the idea that lung cells “remember” past infections, which affects their response to new ones, reflecting the concept of “trained immunity”.

**Supplementary Information:**

The online version contains supplementary material available at 10.1186/s12964-025-02284-y.

## Background

Influenza (flu) viruses are respiratory pathogens with high zoonotic potential estimated to cause over 5 million hospitalizations worldwide per year [1]. Despite the development and annual adjustment of influenza vaccines, high incidences of flu disease are recorded during seasonal epidemics as well as during the most recent swine flu pandemic in 2009 [2]. Influenza A virus (IAV) and influenza B virus are the most common flu subtypes causing influenza-related disease in humans [3]. Importantly, bacterial secondary infections are frequent complications of influenza and are associated with high morbidity and mortality [3]. *Streptococcus pneumoniae* (*S. pn.*) is a Gram-positive bacterial pathogen and a frequent, mostly asymptomatic colonizer of the human nasopharynx. Epidemiological evidence attested *S. pn.* to be one of the major pathogens identified in post-influenza secondary bacterial infections [4, 5]. Experimental data revealed that a variety of pathogen- and host-specific mechanisms, e.g. increased attachment [6], availability of host-derived nutrients [7, 8] or host antiviral immune responses [9, 10] critically drive influenza/pneumococcal co-pathogenesis. Despite intense research over the last two decades, the detailed mechanisms of this co-pathogenesis remain elusive in many points.

*S. pn.* comprises over 94 serotypes, defined by the organo-chemical structure of their polysaccharide capsule. *S. pn.* in its own right causes invasive pneumococcal disease (IPD), a severe respiratory bacterial infection manifesting e.g. as pneumonia, otitis media or meningitis. Most *S. pn.* serotypes asymptomatically colonize the human nasopharynx. Invasive serotypes are generally found less frequently in the nasopharynx of healthy individuals. Mechanistic understanding of the colonizing/invasive potential of *S. pn.* serotypes is however largely missing.

The vast majority of in vivo studies addressing the lethal synergism between influenza viruses and *S. pn.* employ infection models, in which secondary pneumococcal infection is performed during acute viral pneumonia. In this phase of peak susceptibility, pulmonary integrity is greatly compromised by viral replication as well as antiviral immune responses. However, we and others have previously shown that influenza-mediated susceptibility to *S. pn.* can persist after viral clearance has been accomplished, i.e. during the post-influenza recovery period [11, 12]. In contrast to equal hypersusceptibility to both invasive and colonizing strains during acute influenza infection, we found persisting susceptibility to *S. pn.* only for invasive isolates in resolving IAV infection. Physiologically, this phase is characterized by gradual contraction of antiviral immune effectors including lung innate and adaptive leukocyte subsets and airway inflammatory cytokines (own unpublished data) and proliferation of alveolar type II epithelial cells (AECII) [13]. In the lower airways, AECII together with AECI comprise the epithelial barrier, with a subset of AECII acting as their progenitor cells [14]. AECII are estimated to make up 60% of the alveolar lining cells but cover only about 5% of the alveolar surface [15]. With respect to IAV, AECII are primary sites of viral replication in the alveoli [16]. They substantially shape immune responses against influenza and other respiratory viruses including poxvirus [17] and SARS-CoV-2 [18] upon recognition of viral pathogen-associated molecular patterns (PAMPs) [19] as well as antiviral alarmins such as type I interferons (IFNs). Thereby, AECII, next to other long-lived locally residing cells such as macrophages and dendritic cells (DC), contribute to the sentinel system of the lower airways. Especially in contrast to macrophages [20], little is known about AECII-specific processes in the post-IAV recovery phase and how they may interfere with immediate-early recognition and defense responses to secondary bacterial invaders such as *S. pn.*

Inflammatory priming and trained innate immunity describe mechanisms of sustained imprinting of secondary immune responses by primary triggers. These can be mediated by epigenetic, transcriptional and metabolic changes to resident (immune) cells - including structural cells - and their progenitors, altering their immunological responsiveness [21]. To date, it remains unclear whether and how such mechanisms contribute to sustained enhanced susceptibility to *S. pn.* following influenza infections, especially with regard to structural cells such as AECII.

Here, we employed a murine model of post-influenza secondary pneumococcal infection to analyze *S. pn.* serotype-specific AECII-responses in the regenerating lung with the aim to determine their possible contribution to aberrant antimicrobial immune responses during secondary pneumococcal pneumonia. We confirmed that resolving IAV infection enhances susceptibility to pneumococci in a strain-dependent manner, partly associated with the emergence of a serotype-specific hyperinflammatory airway microenvironment. On the AECII transcriptional level, we found that abrogation of post-flu AECII epithelial repair was a general consequence of pneumococcal secondary infection. AECII from IAV-affected lungs furthermore mounted faster and intensified responses to type I and II interferons induced by the secondary *S. pn.* infection and showed epigenetic adaptations.

## Methods

### Mice

For all experiments, female C57BL/6JOlaHsd mice (age 10–14 weeks, purchased from Envigo at age 8–10 weeks) were used. All obtained mice were specific-pathogen free (SPF), as stated by the supplier’s health certificates. Animals were housed in the animal facility of the Helmholtz Centre for Infection Research under SPF conditions and in accordance with national and institutional guidelines. The in vivo study protocol was reviewed and approved by institutional and regional ethical bodies (Niedersaechsisches Landesamt fuer Verbaucherschutz und Lebensmittelsicherheit).

### Influenza infection

Madin-Darby canine kidney cell-derived IAV (H1N1, PR/8/34, mouse-adapted) was obtained [22] and the 50 % tissue culture infectious dose (TCID_50_) was determined as previously described [23]. Mice were anesthetized by intraperitoneal (*i.p*.) injection of ketamine/xylazine and were intranasally infected with 7.9 TCID_50_ in 25 µL of PBS. Control animals received PBS only.

### Pneumococcal infection

For *S. pneumoniae* infections, the serotype 4 strain TIGR4 (ATCC BAA-334) [24], a serotype 19F strain (BHN100) [25] and a serotype 7F strain (BHN54) [25] were used. Strains were obtained from B. Henriques-Normark (Karolinska Institutet, Stockholm, Sweden). Bacteria were plated from frozen glycerol stocks onto Columbia blood agar plates (BD) and incubated for 12 – 18 h (37 °C, 5 % CO_2_). Single colonies were suspended in fresh Todd-Hewitt yeast medium and the bacterial suspension was grown to the mid-logarithmic phase (OD_600 nm_ ~ 0.4), bacteria were spun down, washed once and diluted in PBS to obtain the final inoculum for infection. CFU were retrospectively determined by plating. For bacterial infections, mice were anesthetized by *i.p*. injection of ketamine/xylazine and 40 µL of the pneumococcal inoculum (or PBS) were administered by oropharyngeal aspiration.

### AECII isolation

Isolation of primary murine AECII was conducted according to a previously established protocol [26] with slight modifications. Briefly, lung single cells suspensions were obtained by enzymatic (dispase / DNase) and mechanical dissociation of whole lungs. Pooled cells from *n* = 3 – 7 mice/cohort were stained with PE- or APC-conjugated antibodies (from either BD or BioLegend) against murine F4/80 (clone: BM8), CD93 (clone: AA4-1), CD11c (clone: N418), CD19 (clone: 6D5), CD31 (clone: 390), CD11b (M1/70), CD16/32 (clone: 2.4G2) and CD45 (clone: 30-F11). AECII were sort-purified by negative selection (PE^ꟷ^, APC^ꟷ^, autofluorescence^hi^, side-scatter^hi^) using FACS Aria II and Fusion instruments (BD) and maximum purity masks (4-way purity mode). FACS-based re-analyses of sorted AECII samples were routinely performed and revealed ~ 95% purity (Additional file 1).

### Generation of bronchoalveolar lavage fluid (BALF)

At experimental endpoints, mice were euthanized, the trachea was exposed and an indwelling venous catheter was inserted. The lungs were flushed with 1 mL ice-cold PBS via the catheter using a syringe. Where appropriate, obtained BALF was used for CFU quantification, then spun down and BALF supernatants were stored at -80 °C for further analyses.

### Colony forming unit quantification

At experimental endpoints, serial dilutions of BALF and blood samples of *S. pn.*-infected mice were plated onto Columbia blood agar plates (BD) to assess airway bacterial burden and bacteremia.

### Albumin, cytokine and chemokine quantification in BALF

Airway serum albumin was quantified by ELISA using BALF samples and anti-mouse albumin coating- and HRP-conjugated anti-mouse albumin detection antibodies (both Bethyl Laboratories) and a purified mouse serum albumin standard (Sigma Aldrich). Airway cytokines and chemokines were quantified by multiplex analyses of BALF samples using LEGENDplex^™^ kits (BioLegend). Cytokine and chemokine concentrations were z-score-transformed, hierarchically clustered and color-coded. Quantification of airway bioactive type I/III IFN was conducted by treating type I/III IFN-sensitive cells isolated from Mx2-Luc reporter mice for 24 h with diluted BALF. Relative IFN levels were determined by quantifying relative light units (RLU) in cell lysates after addition of luciferin-reaction buffer [27]. IFN-α was quantified using the VeriKine-HS^™^ Mouse Interferon Alpha All Subtype ELISA Kit (PBL Assay Science^™^). IFN-β and IFN-λ2/3 were quantified using the respective Mouse DuoSet ELISA kits (R&D Systems^™^).

### Western blot

FACS-sorted, pooled AECII were lysed in SDS sample buffer (4 % SDS, 1.5 M Tris-HCl, 33 % Glycine, 6 % β-mercaptoethanol, 0.5 % bromophenol blue) at 95 °C for 3 – 5 min. Cell lysates were loaded on 10 % acrylamide gels, proteins were separated and blotted on PVDF membranes. The following RRID-listed primary antibodies were used for detection: anti-IRF1 (AB_631838), anti-IRF3 (AB_2264929), anti-IRF7 (AB_1125072), anti-IRF9 (AB_2296227), anti-IL10RB (AB_1512256), anti-IFNGR2 (AB_2248682), anti-STAT1 (AB_2198300), anti-STAT2 (AB_2799824), anti-vinculin (AB_2819348) and anti-GAPDH (AB_2107448). HRP-conjugated secondary antibodies reactive against either mouse, rabbit, goat or hamster Ig were utilized. Blots were developed using Lumi-Light western blotting substrate (Millipore) and imaged using the INTAS ECL Chemocam Imager (Intas Science Imaging). Average band intensities were normalized against the respective loading controls using ImageJ software.

### Transcription analysis

Total RNA from FACS-sorted, pooled AECII (*n* = 3 independent replicates per experimental condition, cells pooled from *n* = 3 – 5 mice / replicate) was isolated using the RNeasy Plus Mini Kit (Qiagen). Clariom S microarray analysis (Affymetrix, Thermo Fisher Scientific) was performed at the Helmholtz Center for Infection Research (Braunschweig, Germany) according to manufacturer’s recommendations. Raw data were analyzed with the Transcriptome Analysis Console (Thermo Fisher Scientific) using the SST-RMA algorithm. Transcripts with log_2_ signal intensities (SI) below the 20th percentile of the overall SI-distribution in all 42 microarrays were excluded from further analysis. Differential gene expression was calculated versus the mean of the PBS control replicates. Transcripts with a fold change (FC) of|FC| > 3 and ANOVA-based p-value of *p* < 0.05 were considered significant. Normalized log_2_ SI data of these transcripts were further processed to infer a gene co-expression network based on the ARACNE algorithm [28], implemented in the Cyni Toolbox add-on [29] of the Cytoscape analysis software [30]. The ARACNE algorithm settings were as follows: Mutual information (MI) calculation was performed using the adaptive partitioning option [31]. DPI tolerance was set to 0. As a regulatory basis for the ARACNE network, a list of all known murine transcription factors was used, that was compiled from online resources from the Gene Ontology Consortium. The MI-threshold was empirically set to 0.6, resulting in a network with 449 nodes and 791 edges with three network components. Minor unconnected network components as well as unconnected individual nodes were excluded from further analyses. The resulting ARACNE gene co-expression network was further analyzed using Gephi software [32] for node arrangement by multi-gravity force atlas algorithm [33], modularity/spectral network partitioning [34] and calculation of basic network characteristics like node connectivity, clustering coefficient and node betweenness centrality. For the visualization and plotting of network figures and associated characteristics, a series of Python scripts was used. For ANOVA analysis, visualization and clustering of gene expression and gene ontology results, Genesis software [35] was used. Gene ontology analysis was performed using the ClueGO plugin for Cytoscape Software. For receptor/ligand mapping, the database from CellTalkDB [36] together with a Python script was used. Microarray data were deposited online and are available at NCBI’s Gene Expression Omnibus (GEO) under reference ID: GSE225343. Python scripts for the analysis and visualization of transcriptomic data and network representation are available upon reasonable request.

Further, we constructed a network based on the “Algorithm for the Reconstruction of Accurate Cellular Networks” (ARACNE) [28, 31, 37]. ARACNE aims at identifying direct transcriptional interactions by inferring pair-wise mutual information (MI) between regulated genes and subsequent efficient pruning of distracting and likely indirect correlations, commonly arising in cascades of regulatory transcriptional interactions and their feedback responses. Thereby, ARACNE generates a graph representation of the gene-regulatory network topology in which genes are represented as nodes that are connected by edges, representing potential direct regulatory interactions.

### Assay for transposase-accessible chromatin with sequencing

FACS-sorted AECII (pooled from 5 – 6 mice/experimental group) were frozen in culture medium containing FBS and 5 % v/v DMSO. Cryopreserved cells were sent to Active Motif Inc. for ATAC-sequencing. Cells were thawed in a 37 °C water bath, pelleted, washed with cold PBS, and tagmented as previously described [38], with some modifications based on Corces et al. (2017) [39]. Briefly, cell pellets were suspended in lysis buffer, pelleted, and tagmented using the enzyme and buffer provided in the Nextera Library Prep Kit (Illumina). Tagmented DNA was purified using the MinElute PCR purification kit (Qiagen), amplified with 10 cycles of PCR, and purified using Agencourt AMPure SPRI beads (Beckman Coulter). Resulting material was quantified using the KAPA Library Quantification Kit for Illumina platforms (KAPA Biosystems) and sequenced with PE42 sequencing on the NextSeq 500 sequencer (Illumina). Reads were aligned using the BWA algorithm [40] (mem mode; default settings). Duplicate reads were removed, only reads mapping as matched pairs and only uniquely mapped reads (mapping quality ≥ 1) were used for further analysis. Alignments were extended in silico at their 3’-ends to a length of 200 bp and assigned to 32-nt bins along the genome. The resulting histograms (genomic “signal maps”) were stored in bigWig files. Narrow peaks were identified using the MACS 2.1.0 algorithm at a cutoff of p-value 10^− 7^, without control file, and with the “–nomodel” option. Peaks that were on the ENCODE blacklist of known false ChIP-Seq peaks were removed. Signal maps and peak locations were used as input data to Active Motifs proprietary analysis program, which creates Excel tables containing detailed information on sample comparison, peak metrics, peak locations and gene annotations. For differential analysis, reads were counted in all merged peak regions (using Subread), and the replicates for each condition were compared using DESeq2 [41]. Analysis was conducted using the following software: bcl2fastq2 (v2.20) for processing of Illumina base-call data and demultiplexing, Samtools (v0.1.19) for processing of BAM files, BEDtools (v2.25.0) for processing of BED files, wigToBigWig (v4) for generation of bigWIG files and Subread (v1.5.2) for counting of reads in BAM files for DESeq2 and HOMER motif analysis tool. ATAC-regions with|shrunken log_2_ FC| > 0.25 (IAV day 14 vs. PBS control) and an adjusted p-value < 0.1 were considered significant. Additional analysis and data visualization was performed using the Deeptools library [42], self-written Python scripts and IGV Genome Browser [43]. Gene ontology analysis of gene loci with differential ATAC-regions was performed using the ClueGO plugin for Cytoscape Software. ATAC-seq data were deposited online and are available at NCBI’s Gene Expression Omnibus (GEO) under reference ID: GSE225498.

## Results

### Susceptibility to secondary *S. pn.* infection following IAV infection depends on the pneumococcal serotype

**Fig. 1:**
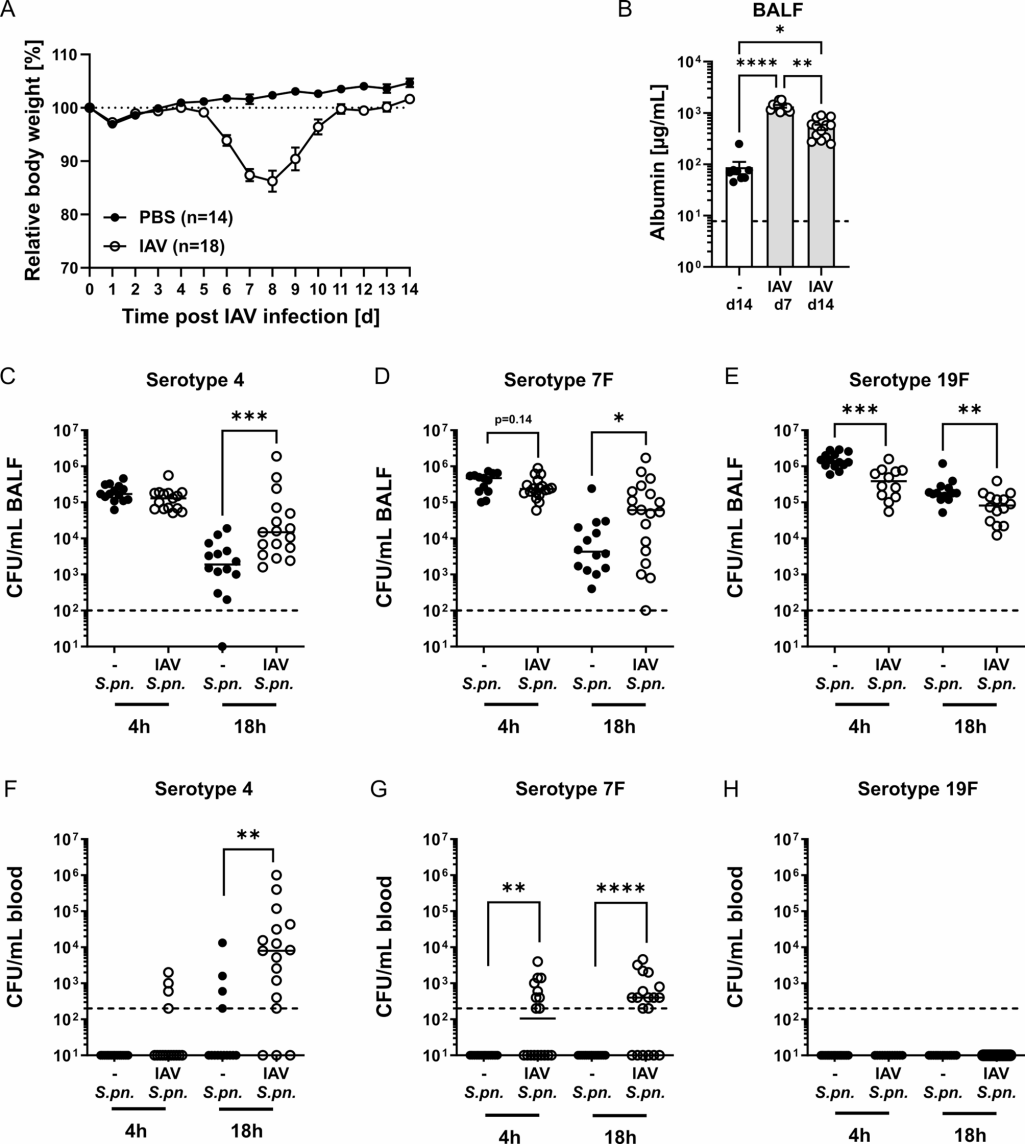
Influenza A virus infection sustainably decreases lung barrier integrity and alters susceptibility towards *Streptococcus pneumoniae*. Mice were intranasally infected with 7.9 TCID_50_ IAV (H1N1, PR/8/34) or treated with PBS. **(A)** Body weight of PBS- and IAV-treated mice. Mean % body weight (relative to baseline at d 0) ± SEM is graphed. Data were compiled from 3 independent experiments with 3 – 7 mice/group/experiment. **(B)** Serum albumin in bronchoalveolar lavage fluid (BALF). Data were compiled from 2 independent experiments with *n* = 4 – 7 mice/group/experiment. Mean (bars) ± SEM and individual values are depicted. The dashed line indicates the detection limit. Statistical analysis was performed by Kruskal-Wallis and Dunn’s multiple comparisons test, *p < 0.05, *p* < 0.01, **p < 0.0001. At day 14 post primary treatment, IAV- and PBS-treated mice were oropharyngeally infected with 10^6^*S. pn.* (serotype 4, 7F or 19F). Pneumococcal colony forming units (CFU) in BALF **(C**,** D**,** E)** and blood **(F**,** G**,** H)** were assessed at 4 – 18 h post bacterial infection. Statistical analysis was performed by two-tailed Mann-Whitney (C, D,E) or Fisher‘s exact test (F, G,H), *p* < 0.05, *p* < 0.01, *p* < 0.001, ****p < 0.0001

To analyze sustained effects of IAV infection on AECII antibacterial responses, we employed a previously established murine infection model. Intranasal infection of C57BL/6 mice with a sublethal dose of a strictly pneumotropic, mouse-adapted strain of IAV (PR/8/34, H1N1) resulted in acute viral pneumonia [12]. IAV-mediated disease included moderate and, transient weight loss (Fig. 1A) and disruption of the pulmonary barrier, demonstrated by significantly increased levels of serum albumin in the bronchoalveolar space 7 and 14 days post infection as compared to controls (Fig. 1B). Of note, intra-alveolar leakage of serum albumin was most pronounced during acute infection (day 7 post IAV) corresponding with peak lung viral titers (Additional file 2 and Additional file 13) and significantly decreased during the post-IAV recovery phase (day 14) as compared to acute infection. At day 14 after IAV infection, post-IAV and control mice were infected with one of three strains of *Streptococcus pneumoniae* (*S. pn.*) of serotypes 4, 7F and 19F, differing in their invasive disease potential (4 > 7F > 19F). Taking into account previously reported influenza-mediated changes in the upper respiratory tract of humans [44] and mice [12, 45], which might affect bacterial translocation into the lower airways, pneumococcal inocula were administered by oropharyngeal aspiration. At 18 h post pneumococcal infection, the airway bacterial burden was significantly increased in mice infected during resolving IAV infection for the *S. pn.* serotype 4 (Fig. 1C) and 7F (Fig. 1D) strains as compared to the respective *S. pn.* infection alone. In stark contrast, decreased airway CFU were detected already at 4 h as well as 18 h post *S. pn.* 19F infection in post-IAV mice as compared to 19F infection alone (Fig. 1E). In line with increased airway bacterial loads of the *S. pn.* serotype 4 and 7F strains in mice infected post-flu, an increased incidence of bacteremia with significantly increased mean CFU in the blood was observed after secondary *S. pn.* infection with these strains (Fig. 1F and G). *S. pn.* 19F generally did not cause bacteremia (Fig. 1H).

Taken together, *S. pn.* outgrowth in the respiratory tract and dissemination were enhanced in post-IAV mice, strongly depending on the *S. pn.* serotype. Serotype 4 showed the most invasive potential already in *S. pn.* single infection, with its invasiveness drastically increasing in previously IAV-infected mice. Invasiveness of serotype 19F was not affected by preceding IAV infection with even lower respiratory CFU in this scenario. Serotype 7F, which in *S. pn.* infection alone was not invasive within 18 h of the bacterial infection, however crossed the lung mucosal barrier and disseminated via the bloodstream when infection was preceded by IAV infection. These results clearly demonstrate serotype-dependent mechanisms at play in the synergism between infections with IAV and *S. pn.* Here, the IAV/7F infection model stood out with a strong IAV-mediated effect on bacterial invasiveness.

### The airway cytokine response towards *S. pn.* infection is significantly altered by previous IAV infection and between pneumococcal serotypes

**Fig. 2:**
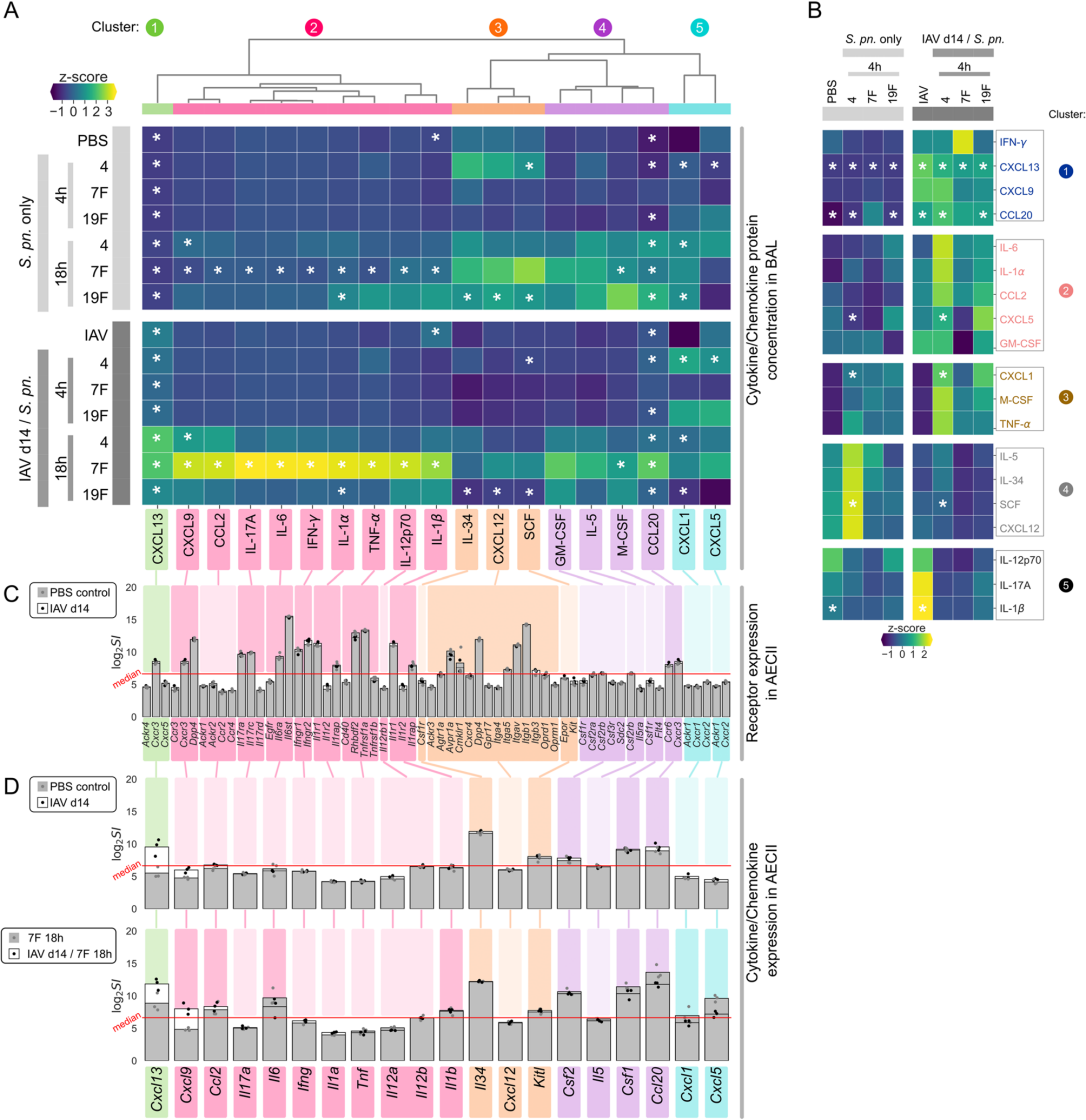
Resolving influenza A virus infection alters airway cytokine and chemokine levels upon *Streptococcus pneumoniae* infection. Mice were intranasally infected with 7.9 TCID_50_ IAV (H1N1, PR/8/34) or treated with PBS. On day 14 post infection (*p.i.*), IAV-infected and PBS-treated mice were oropharyngeally infected with 10^6^*S. pn.* (serotype 4, 7F or 19F). Cytokine/chemokine concentrations in bronchoalveolar lavage fluid (BALF) were assessed at 4 – 18 h post bacterial infection. Data were compiled from 1 – 3 independent experiments with *n* = 2 – 7 mice/group/experiment, such that each analyte was covered by 3 – 19 (average: 11) data points per group. **A)** Z-scores of mean cytokine/chemokine concentrations of each experimental group were used for heatmapping and clustering (cluster 1 – 5). Data for only the early (4 h) BALF cytokine/chemokine levels **(B)** are additionally shown. IAV/*S. pn.* conditions were compared to the respective *S. pn.* only condition and IAV only condition was compared to PBS control, with a two-sided Mann-Whitney-U test. Asterisks indicate *p < 0.05. **C)** Gene symbols of known receptors to clustered BALF cytokine/chemokines were obtained from the receptor/ligand database CellTalk DB. AECII receptor expression as log_2_ signal intensity (log_2_ SI) for PBS control and IAV 14 days *p.i.* was derived from AECII microarray analysis (*n* = 3 per group). Bars represent mean log_2_ SI with grey and black points indicating values of microarray replicates. Receptors with expression above median signal intensity of the microarray’s total SI-distribution (red line) are highlighted by the according BALF cytokine/chemokine cluster color. **D)** AECII expression levels of BALF cytokine/chemokine genes matching the respective receptors and cytokine/chemokine proteins in the BALF were derived from microarray data for 7F 18 h single infection and IAV/7F 18 h secondary infection. Gene expression above median SI is highlighted by the according BALF cytokine/chemokine cluster color

To address possible associations between the strain-dependent impaired clearance of *S. pn.* following IAV infection and the airway inflammatory microenvironment, we quantified 19 cytokines and chemokines in BALF from mock- and IAV-infected mice as well as mock-/*S. pn.-* and IAV/*S. pn.*-infected mice 4 h and 18 h after the secondary challenge (Fig. 2A). To better visualize immediate-early responses, only the cytokine/chemokine levels detected at the 4 h time-point are presented in Fig. 2B.

Comparison of BALF from only IAV-infected to PBS-treated mice revealed a small, significant, increase in the inflammatory mediators CXCL13, IL-1β and CCL20 (Fig. 2A and Additional file 3). Following *S. pn.* infection alone, highest cytokine/chemokine levels were observed after 18 h. With respect to *S. pn.* following IAV infection, similar levels of GM-CSF, IL‑5 and CXCL5 were detected in the airways of *S. pn.*- and IAV/*S. pn.-*infected mice. At the same time, previous IAV infection partly blunted the production of IL-34, CXCL12 and SCF upon secondary *S. pn.* infection with most serotypes (Fig. 2A). Immediate-early pro-inflammatory responses 4 h post IAV/*S. pn.* infection showed subtle and partly strain-specific signatures (Fig. 2B, clusters 1 –3). While previous IAV infection led to significantly increased early (4 h) induction of CXCL13, CCL20, CXCL5 and CXCL1 upon secondary infection with *S. pn.* serotype 4 (Fig. 2B), it particularly favored the development of a hyperinflammatory microenvironment during secondary infection with *S. pn.*  7F at 18 h post pneumococcal infection (Fig. 2A). This included significantly increased levels of several cytokines associated with type 1 (TNF-α: 12-fold increase, IL-12: 4-fold increase, IFN-γ: 123-fold increase) and type 3 (IL-17A: 20-fold increase) immune responses. Of note, IFN-γ showed the by far strongest induction (Fig. 2A). Also, protein concentrations of CXCL13, CXCL9, CCL2, IL-6, IL-1α, IL-1β, M-CSF and CCL20 were significantly increased in the BALF IAV/7F secondary infection (Fig. 2A). These observations were well in line with the strain-specific impairment of bacterial clearance and containment in resolving IAV infection that had been particularly evident for strain 7F (Fig. 1).

The overall aim of our study was to generate insight into the serotype-specific modulation of AECII-responses to *S. pn.* in secondary pneumococcal infection and data on bacterial loads and cytokine/chemokine responses in IAV-, *S. pn.*- and IAV/*S. pn.* infection provided the basis for these analyses. To test the responsiveness of AECII to the detected airway cytokine/chemokine milieu at baseline and 14 days post IAV infection, we analyzed AECII transcriptomic data from mock- and IAV-infected mice for transcription of the corresponding receptor genes employing the CellTalk receptor/ligand database (Fig. 2C). Not all known receptor genes analyzed were expressed above the median signal intensity level (average signal distribution is shown in Additional file 4), but AECII isolated from mock- and IAV-infected mice showed relevant expression of receptors for CXCL13 (*Cxcr3*), CXCL9 (*Cxcr3* and *Dpp4*), IL-17a (*Il17ra* and *IL17rc*), IL-6 (*Il6ra* and *Il6st*), IFN-γ (*Ifngr1* and *Ifngr2*), IL-1α and IL-1β (*Il1r1* and *Il1rap*), TNF-α (*Rhbdf2* and *Tnfrsf1a*), CXCL12 (*Avpr1a*, *Cmklr1*, *Dpp4*, *Itga5*, *Itgav*, *Itgb1* and *Itgb3*) and CCL20 (*Ccr6*, *Cxcr3*). Interestingly, AECII gene transcription of receptors to the cytokines/chemokines quantified in BAL was similar when comparing mock- and IAV-infected mice.

Next to the expression of cytokine and chemokine receptors and responding to their signals, AECII are known sources for many of the BALF cytokines and chemokines detected on the protein level [17, 19, 46] and we asked whether this was altered in secondary pneumococcal infection. We analyzed sustained transcription of the genes for the detected cytokines and chemokines in AECII following IAV infection alone as compared to mock infection as well as their transcription in IAV-/*S. pn.* infection as compared to *S. pn.* infection alone (Fig. 2D). The latter analysis was performed exemplarily for 7F- and IAV/7F infection (18 h post *S. pn.*), as this serotype had shown the strongest IAV-dependent modulation of the respiratory cytokine/chemokine response. At steady state (day 14, PBS control), AECII only expressed few of the tested BALF-mediators above median expression level (*Il34*, *Kitl*, *Csf2*, *Csf1* and *Ccl20*). Further, *Cxcl13* was the only gene that showed clearly increased expression in AECII in resolving IAV infection as compared to the mock-infected control (Fig. 2D, upper panel). Comparing 7F infection alone to IAV/7F infection, expression of *Cxcl13* and *Cxcl9* was markedly increased in AECII upon secondary infection (Fig. 2D, lower panel). With respect to the strong respiratory cytokine/chemokine response detected following IAV/7F infection, AECII presumably made only minor contributions and the mediators detected at high concentrations in the BALF (cytokine/chemokine cluster 2) were likely of different cellular origin. Nevertheless, we found AECII to express receptors mediating responses to these inflammatory mediators. Based on these findings in the AECII transcriptional response to IAV, *S. pn.* 7F and *S. pn.*  7F after IAV infection, we performed comprehensive analyses of AECII gene transcription following infections with the three different *S. pn.* serotypes alone or in secondary infection during resolving IAV infection.

### ARACNE gene co-expression network analysis dissects AECII transcriptional response to interferons

Overall, we isolated triplicate AECII samples from the lungs of mice infected with *S. pn.* serotypes 19F, 7F or 4 on day 14 following PBS-treatment or IAV infection at 4 and 18 h post the bacterial challenge. Reference groups were treated with PBS or infected with IAV only. This approach resulted in 14 experimental conditions: (1) PBS, (2) IAV day 14, (3) 19F 4 h, (4) 19F 18 h, (5) 7F 4 h, (6) 7F 18 h, (7) 4 4 h, (8) 4 18 h, (9) IAV/19F 4 h (10) IAV/19F 18 h, 11.) IAV/7F 4 h, 12.) IAV/7F 18 h, 13.) IAV/4 4 h, 14.) IAV/4 18 h. Mice only infected with *S. pn.* were treated with PBS 14 days prior infection to reflect effects of the vehicle solution and application procedure. Differential gene expression was calculated in reference to AECII from PBS-treated mice. From all conditions, 1,037 genes with an absolute transcriptional regulation fold-change of above 3 and an ANOVA-based p‑value < 0.05 were identified (Additional file 5).

**Fig. 3:**
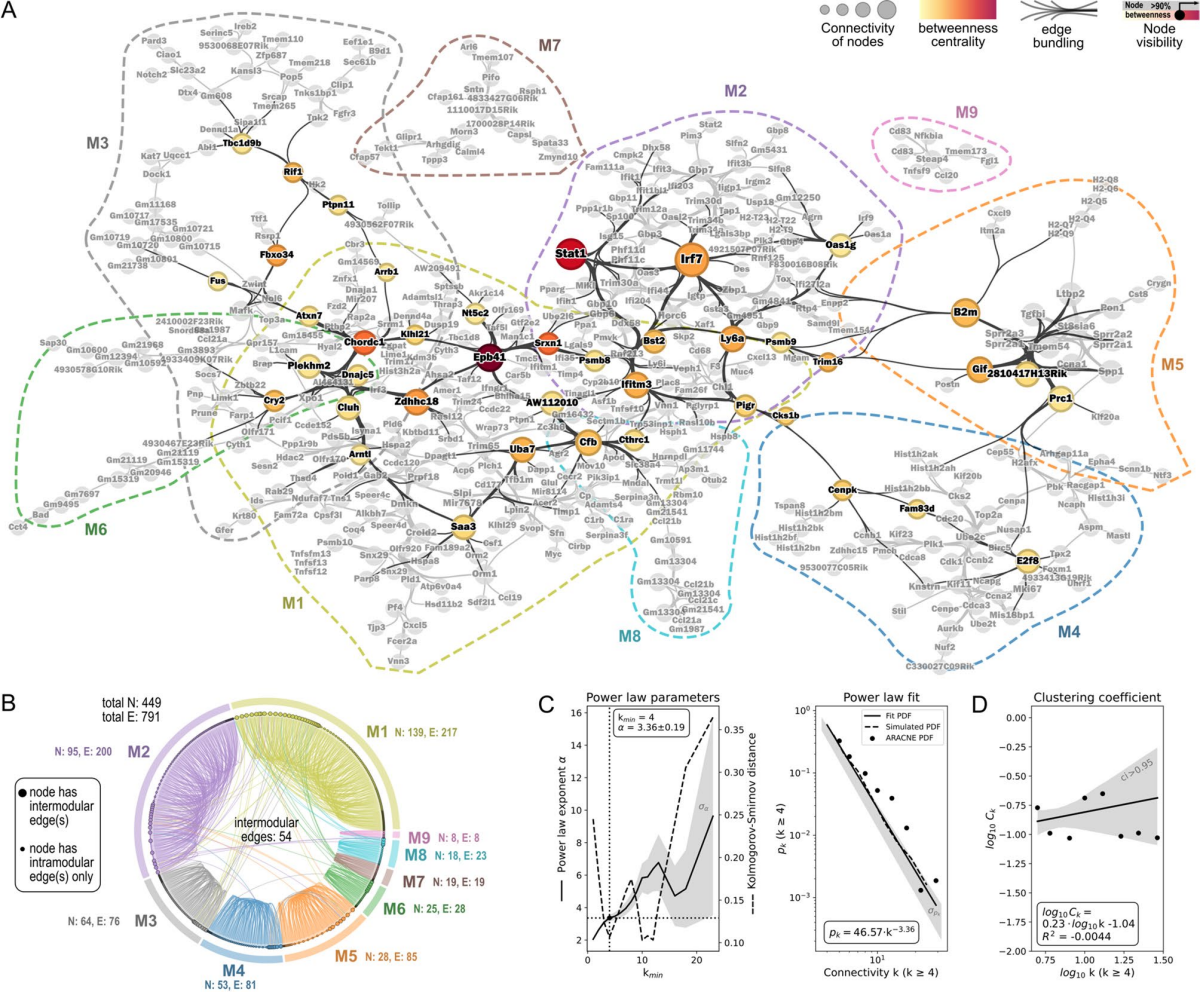
AECII ARACNE gene co-expression network inference. Mice were intranasally infected with 7.9 TCID_50_ IAV (H1N1, PR/8/34) or treated with PBS. On day 14, IAV-infected and PBS-treated mice were oropharyngeally infected with 10^6^*S. pn.* (serotype 4, 7F or 19F). AECII were isolated 4–or 18 h post bacterial infection from pooled lung cells (*n* = 3 – 5 mice per group) from 3 replicate experiments. Differential gene expression between the mean expression per condition and the mean of PBS control AECII was assessed. Genes with fold changes (FC) with|FC| > 3 in at least one condition were tested for significance by ANOVA (*p* < 0.05). Normalized log_2_ SI data of significantly regulated genes were analyzed by the ARACNE gene co-expression network inference algorithm. **(A)** The ARACNE network was partitioned using modularity/spectral partitioning into nine modules M1 – M9 (colored dashed lines) and visualized following multi-gravity force-atlas node positioning. Node size: Node connectivity. Node labels: Gene symbols. Edge visualization was simplified by the hammer edge bundling algorithm. Betweenness centrality of genes was calculated and genes with betweenness centrality > 90th percentile are highlighted. Their betweenness centrality is color-coded and their edges are shown in black. Remaining genes and edges are grayed out. **(B)** Chord plot representation of the ARACNE network and modules. N: number of nodes, E: number of edges. Intra- and inter-modular edges are shown. **(C)** Power law regression fit of node connectivity k. Left: Optimization of fit parameters k_min_ and power law exponent α based on Kolmogorov-Smirnov fitting quality. Right: Best power law fit of p_k_ vs. k for k ≥ 4. p_k_: Binned fractions of nodes with connectivity k. Gray areas represent variance (σ) of α and p_k_, respectively. **(D)** Clustering coefficient distribution characteristic of nodes with k ≥ 4. C_k_: Binned fractions of nodes with clustering coefficient C and connectivity k. Gray area represents the 95 % confidence interval

To gain mechanistic insights into the AECII transcriptional responses associated with the different infection scenarios, we constructed an ARACNE gene co-expression network from these genes. Additional file 6 provides node and edge information of the resulting network. ARACNE gene co-expression inference resulted in a network of 449 genes and 791 mutual information (MI) based correlations (Fig. 3A). The five genes with the highest node connectivity k were transcription factors *Irf7* (k = 29, Interferon regulatory factor 7) and *Stat1* (k = 23, Signal transducer and activator of transcription 1) as well as the genes *B2m* (k = 18, Beta-2 microglobulin), *Epb41* (k = 16, Erythrocyte membrane protein band 4.1) and *Ly6a* (k = 13, Lymphocyte antigen 6 complex locus A). The network was partitioned into nine distinct modules (termed: M1 - M9, Additional file 7), with modules 1 and 2 containing most genes (M1: 139 genes, M2: 95 genes) and thus dominating the network. Dominance of M1 and M2 not only related to the number of genes but also to their intra- and inter-modular connectivity, as e.g. evidenced by the number of genes in M1 and M2 with intermodular connections (Fig. 3B). Of note, though M1 contained 44 genes more than M2, both modules had similar edge counts (M1: 217 edges, M2: 200 edges), indicating that genes in M2 were much more densely connected within their module than genes in M1. Figure 3A additionally gives a qualitative impression on the major hub genes of the network. This is based on the highest node betweenness centrality measure (> 90th percentile). Betweenness centrality was highest for *Epb41*,* Stat1*,* Srxn1* (Sulfiredoxin 1), *Chordc1* (Cysteine and histidine rich domain containing 1), *Zdhhc18* (Zinc finger DHHC-type palmitoyltransferase 18), *Fbxo34* (F-box protein 34), *Irf7*,* Ly6a*,* Ifitm3* (Interferon induced transmembrane protein 3) and *Trim16* (Tripartite motif containing 16), revealing these genes to play a central role in the AECII gene co-regulatory network. We classified the obtained ARACNE network to be of scale-free-like nature, based on log_10_ p_k_ vs. log_10_ k power law fit of the node connectivity distribution (Fig. 3C, left). The optimal power law fit for k ≥ 4 (Fig. 3C, right) had an absolute value of 3.36 ± 0.19 for the exponent α, with genuine scale-free networks based on the Barabási–Albert model having power law exponents between 2 and 3 [47, 48]. In line with this, the ARACNE network’s clustering coefficient distribution in the log_10_ C_k_ vs. log_10_ k plot was independent (R^2^ = ‑0.004) from the node connectivity (Fig. 3D), as could be expected for a network of this type [49]. Considering the scale-free-like topology of our ARACNE AECII-response network, in subsequent analyses we focused on the two apparent hub genes *Irf7* and *Stat1* with the highest node connectivities.

**Fig. 4:**
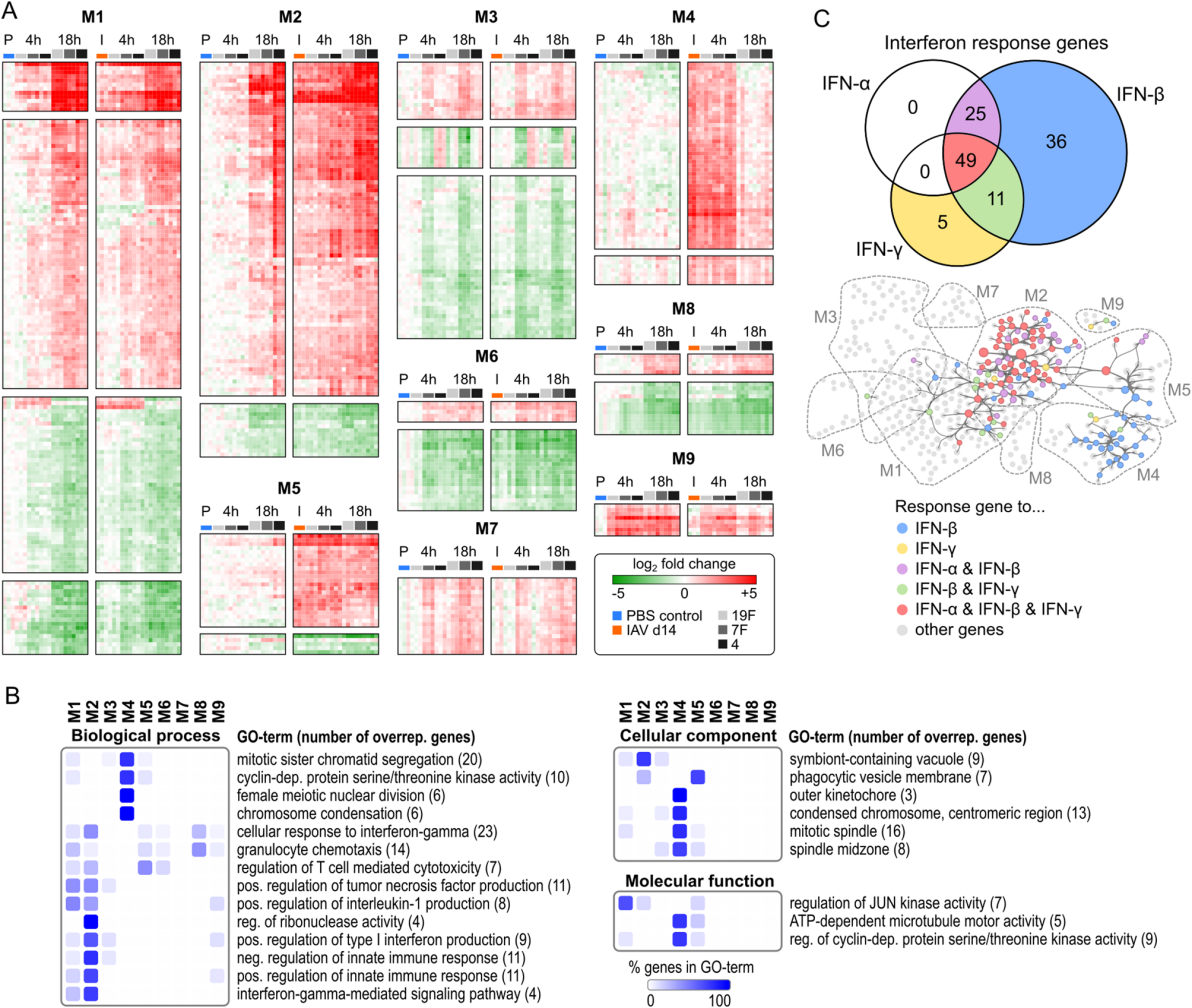
AECII ARACNE network reveals dominance of IFN-triggered responses in resolving IAV infection and acute secondary *S. pn.* infection. **(A)** Clustered and color-coded log_2_ fold changes (over PBS control) of genes in the ARACNE network modules M1 – M9. Left sub-clusters represent PBS (day 14) treatment and *S. pn.* infections alone, right sub-clusters represent IAV infection (day 14) and secondary IAV/*S. pn.* infections with *S. pn.* serotypes 19F, 7F and 4. **(B)** Gene ontology enrichment analysis (two-sided hypergeometric test, FDR < 0.05) of genes in the ARACNE network for indicated GO categories. Genes associated with significant GO-terms were mapped to ARACNE network modules (M1 – M9) and percent of term-associated genes per module were calculated, clustered and color-coded. The total number of network genes per GO-term is stated in brackets. **(C)** Genes in the ARACNE network were analyzed for interferon (IFN-α, IFN-β, IFN-γ) response genes using the Interferome database. The numbers of genes responsive to a specific or multiple IFNs are presented as a Venn diagram. The groups of IFN-response genes, as displayed in the Venn diagram, were mapped to the ARACNE network. Dashed lines represent network module outlines

To understand how genes within the network’s modules were regulated in the different infection scenarios, their log_2_ fold change regulation was clustered and color-coded (Fig. 4A). Functional gene ontology (GO) annotation of genes in the nine modules clearly attributed M1 and M2 to interferon responses and innate immune responses as well as IL-1 and TNF production (Fig. 4B). M1 contained several gene products regulating Jun kinase activity, a biochemical mechanism induced by stress responses leading to assembly of the JUN/FOS (AP-1) transcription factor complex [50, 51]. Interestingly, M3 emerged in the ARACNE network as an especially 7F-related module (Fig. 4A). However, this module was hardly associated with common GO-terms, so that its functional implications remain unclear. M4 clearly attributed to mitosis, proliferation and chromatin organization. Cellular component GO-terms showed genes in M5 to be related to phagocytic vesicles, while M8 was to some extent pointing at granulocyte chemotaxis. Like M3, also the small modules M6, M7 and M9 did not clearly annotate to any common GO-terms, likely due to their low gene count. Nevertheless, a number of genes from M9 (e.g. *Ccl20*,* CD83* and *Nfkbia*) encode for well-known players in the context of acute inflammation [52–54].

GO-term enrichment suggested a major involvement of the interferon system in the AECII response to infection. Therefore we mapped all ARACNE network nodes to known type I (IFN‑α, IFN‑β) and type II (IFN‑γ) interferon response genes using the Interferome database (Fig. 4C) [55]. We set a 3-fold regulation cutoff and limited the response time‑frame to 18 h to match the Interferome database search with our dataset. Out of the 449 genes in the ARACNE network, 126 (28 %) were listed in the Interferome database as responders to type I and/or type II interferon signaling. It is known that the different interferons induce partly overlapping transcriptional responses, hence 49 out of the 126 IFN-response genes (~ 39 %) respond to IFN-α, IFN-β as well as IFN-γ. Of note, 36 genes (~ 29 %) specifically respond to IFN-β, 5 genes were exclusive IFN-γ response genes (~ 4 %) and none of the genes were specific to IFN-α. Further, 25 (~ 20 %) were shared between IFN-α and IFN-β and 11 genes (~ 9 %) between IFN-β and IFN-γ (Fig. 4C top). Mapping the interferon response genes to the ARACNE network revealed a dominant impact on the co-expression network. This was clearly centered within M2, but also reached into M1, M4, M5 and M9 (Fig. 4C, bottom). Of note, M2 IFN-response genes were largely related to all three considered interferons. However, IFN-response genes in M4 were attributed largely to IFN‑β and otherwise annotated to cell proliferation, based on GO-enrichment. This indicated that IFN‑β might be relevant for AECII proliferation or replenishment following pathogenic encounter.

Taken together, we successfully inferred an ARACNE-based scale-free-like gene co-expression network from transcriptional profiles of AECII sorted from mice infected with IAV and one of three serotypes of *S. pn.* with and without previous IAV infection. This network revealed distinct hub-genes in the AECII transcriptional response and was dominated by two out of nine modules (M1 and M2), with M2 appearing heavily related to IFN-response genes.

### AECII mount *S. pn.* serotype-specific transcriptional responses related to differential co-expression network usage

**Fig. 5:**
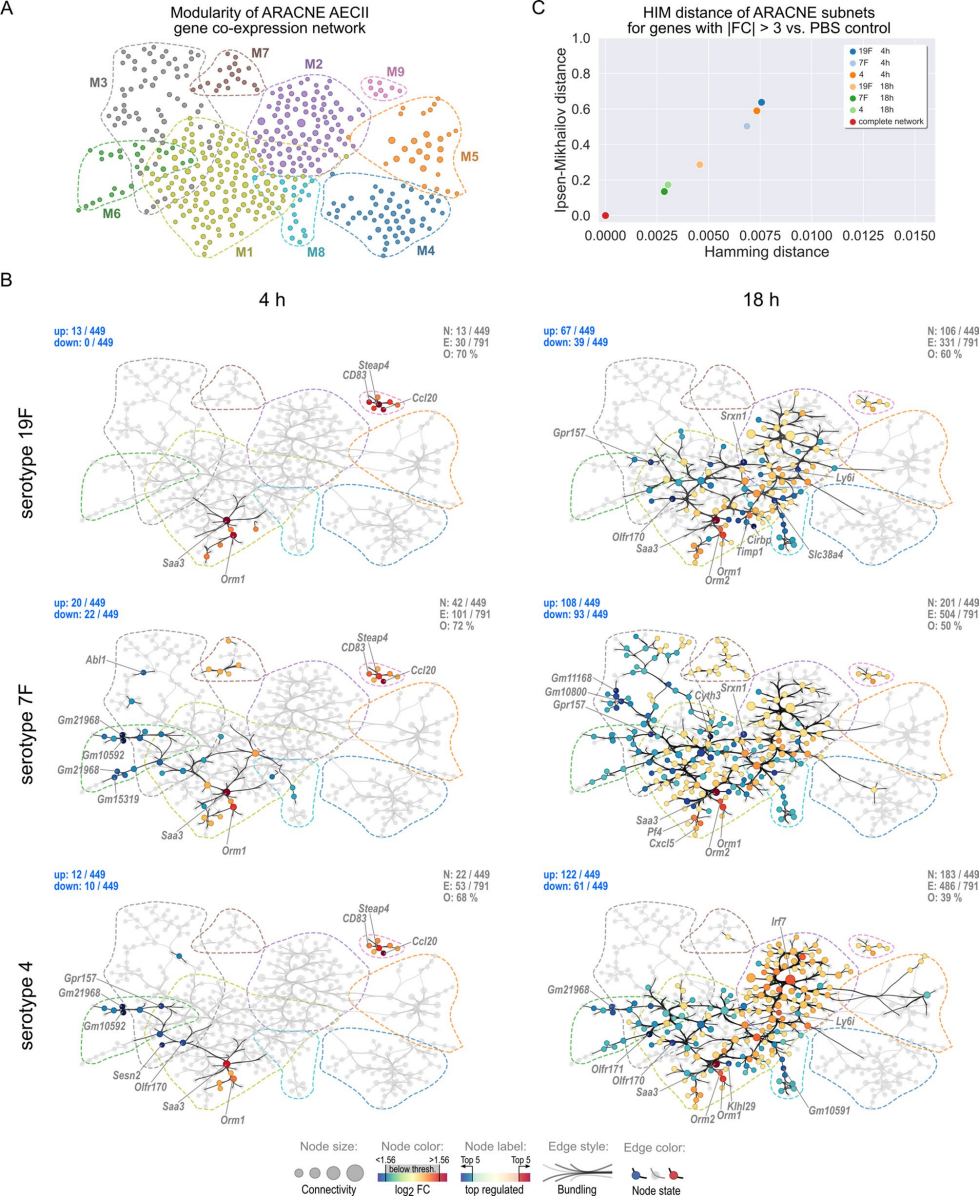
AECII ARACNE differential partial networks reveal serotype-specific network response patterns following *S. pn.* infection alone. **(A)** Module assignment of individual genes to ARACNE network modules M1 – M9. Dashed lines indicate module outlines. **(B)** Partial networks for genes with|FC| > 3 and their associated edges were calculated for *S. pn.* infections with the indicated serotypes alone at 4 h and 18 h post infection. Nodes are color-coded according to log_2_ FC. Remaining nodes and edges are grayed-out. Node size indicates node connectivity. Dashed lines indicate outlines of network modules. Gene symbols of the top 5 most intensely up/down regulated genes are labeled. N: number of nodes with|FC| > 3 vs. PBS control. E: number of highlighted edges. O: Percent of edges that are greyed-out. Numbers of up/downregulated genes are stated in blue in the upper left corners. **(C)** HIM distances of FC-based partial networks shown in B in reference to the complete ARACNE network

**Fig. 6:**
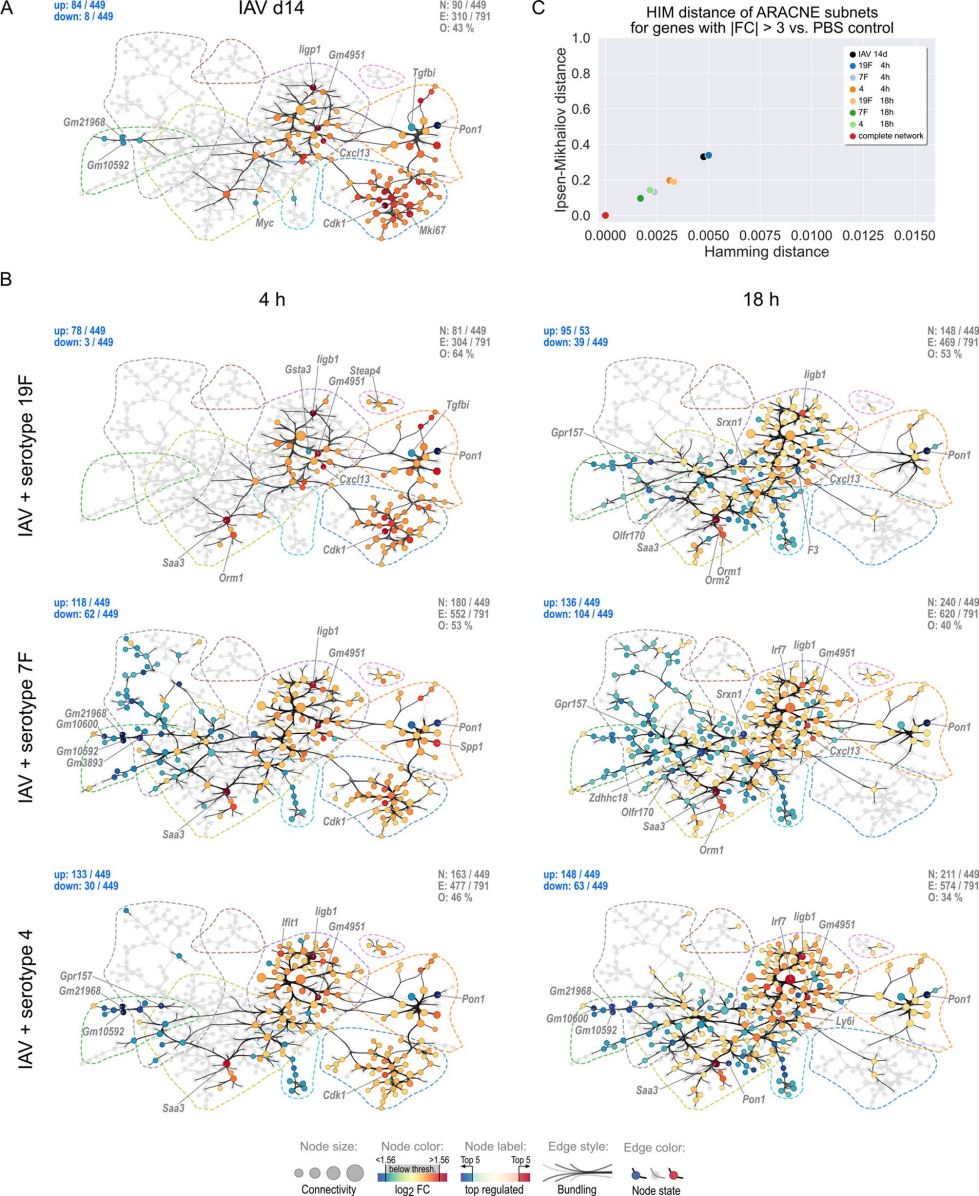
AECII ARACNE differential partial networks reveal IAV-mediated changes in AECII transcriptional regulation and serotype-specific network patterns in secondary *S. pn.* infection. Partial networks for genes with|FC| > 3 and their associated edges were calculated for the AECII gene transcriptional profile detected 14 days post IAV infection **(A)** and 4 h and 18 h following secondary *S. pn.* infection with serotypes 19F, 7F or 4 **(B)**. Nodes are color-coded according to log_2_ FC. Remaining nodes and edges are grayed-out. Node size indicates node connectivity. Dashed lines indicate outlines of network modules. Gene symbols of the top 5 most intensely up/down regulated genes are labeled. N: number of nodes with|FC| > 3 vs. PBS control. E: number of highlighted edges. O: Percent of edges that are greyed-out. Numbers of up/downregulated genes are stated in blue in the upper left corners. **C)** HIM distances of FC-based partial networks shown in B in reference to the complete ARACNE network

We further mapped FC regulation of gene transcription of the different experimental conditions to the ARACNE network. Thereby, we (i) assessed how the topological ARACNE gene co-expression network structure and modularity related to the temporal regulation of gene transcription in AECII upon *S. pn.* infection alone and secondary *S. pn.* infection following IAV infection and (ii) identified serotype-dependent differences in network usage. Network nodes were color-coded according to the log_2_ fold change regulation of the respective transcript or hidden, if the represented gene did not show significant regulation with|FC| > 3. Figure 5A shows the assignment of nodes to the nine network modules M1 – M9. Figure 5B shows the network representations for AECII isolated from only *S. pn.*-infected mice (19F, 7F, 4; 4 h and 18 h). Likewise, Fig. 6 represents the resulting network maps for transcriptional regulation in AECII isolated following IAV infection alone (day 14; Fig. 6A) and secondary *S. pn.* infection following IAV infection (IAV/19F, IAV/7F, IAV/4; 4 h and 18 h; Fig. 6B). Additional file 8 provides high-resolution network representations with full gene symbol labeling.

To compare the associated partial networks in Figs. 5B and 6B, we calculated the Hamming-Ipsen-Mikhailov (HIM) distance of each partial network in reference to the complete ARACNE network (Figs. 5C and 6C). The Hamming distance (H) of two networks evaluates the presence/absence of matching network edges, based on the difference of the two network adjacency matrices and thus reflects local network similarities [56]. In contrast, the Ipsen-Mikhailov (IM) distance is a measure of global topological similarities between two networks, based on spectral comparisons of their adjacency matrices [57]. Interestingly, we found that as early as 4 h after *S. pn.* infection alone, serotype-specific patterns amongst the AECII differential partial networks started to emerge (Fig. 5B, left). Genes in M9 (*Ccl20*, *Steap4*, *CD83*, *Nfkbia*,* Tmem173*, *Fgl1* and *Tnfsf9*) were rapidly upregulated following infection with all serotypes, with a reduction in expression at 18 h (Fig. 5B, right). In M1 *Saa3* and *Orm1* showed a strong early transcriptional response 4 h after infection with any of the three tested *S. pn.* serotypes. With respect to serotype-specific responses, genes in M6, such as *Gm10592*, *Gm21968* (both predicted genes) and *Gpr157*, were strongly downregulated in AECII only following 7F and serotype 4 infection. Also, specifically infection with 7F triggered the upregulation of genes in M7 (e.g. *Arhgdig*, *Arl6*, *Glipr1* and *Calml4*) at 4 h. Generally, AECII did not show intense differential gene regulation with|FC| > 3 within the ARACNE network (19F: 13 up; 7F: 20 up, 22 down; 4: 12 up, 10 down) at 4 h post infection with *S. pn.* alone. This was also reflected in the HIM distances of the partial networks. Here, the three respective partial networks located farthest from the complete network and cumulated relatively close to one another (Fig. 5C).

The AECII transcriptional response to *S. pn.* infection alone had increased drastically at 18 h post infection (19F: 67 up, 39 down; 7F: 108 up, 93 down; 4: 122 up, 61 down) and further encompassed serotype-specific network patterns. Transcriptional regulation (up and down) in M1 greatly increased around genes active at 4 h. Interestingly, specifically 7F infection led to a downregulation of gene expression within M3. Also, most genes in M6 were still downregulated 18 h post infection, which was again not the case for infection with 19F. In contrast, most genes in M8 (e.g. *Ccl21a*, *Ccl21b*, *Ccl21c*) were consistently downregulated in infections with all serotypes. M2 majorly contributed to the structure of the three serotype-specific partial networks 18 h post infection. The invasive serotype 4 *S. pn.* strain clearly triggered the strongest response in this module, inducing strong upregulation of virtually each node in the module. As compared to serotype 4, both, the number of genes regulated above threshold as well as the extent of their regulation were not as prominent for infection with serotypes 19F and 7F. This becomes apparent also from the heatmap view of M2 in Fig. 4A. M2 is dominated by interferon response hub transcription factors *Irf7* and *Stat1* that were highly expressed in all three partial networks 18 h post infection. According to the HIM distances graphed in Fig. 5C, partial networks for AECII transcriptional regulation in response to serotypes 4 and 7F (18 h) presented closer to the complete network and the HIM-distance of the 19F (18 h) partial network was clearly distinct from 4 to 7F infection.

14 days post IAV infection alone, 84 out of 449 ARACNE network genes were still upregulated (> 3-fold) and 8 genes were downregulated. While modules M4 and M5 were not extensively perturbed in response to *S. pn.* infection alone, transcriptional regulation of genes within these modules was a strikingly distinct feature of transcriptional regulation in AECII late after IAV infection alone (day 14; Fig. 6A). Here, transcription of all genes in M4, which were previously found to be related to IFN-β responses and various mitotic mechanisms (Fig. 4B and C), were upregulated. Interestingly, *Cdk1*, *Mki67*, *E2f8*, *Cdc20* and *Ube2c*, most of which are directly related to a proliferative transcriptional configuration, were among the most upregulated genes within M4. M5 contains MHC class I (*B2m*) and II genes and similarly showed strong upregulation of most genes attributed accordingly (*e.g. H2‑Q5*, *H2‑Q6*, *H2‑Q7*, *H2‑Q8*, *H2‑Q9*, *Spp1*, *Prc1* and *Gif*), with the exception of *Tgfbi* and *Pon1*, that both were strongly downregulated. Of note, genes in M5 feature the highest clustering coefficients (Additional file 9) amongst all genes in the network, indicative of intricate correlative connections amongst its constituents. Importantly, also many interferon response genes in M2 (e.g. *Cxcl13*, *Iigp1*, *Gm4951*, *Usp18*, *Trim30a*, *Bst2*, *Ly6i*, *Ly6a* and *Irf7*) were upregulated in AECII on day 14 following IAV infection and transcriptional upregulation of *Cxcl13* was well in line with elevated Cxcl13 BALF protein levels under the same condition (Fig. 1). In M1, only few genes were upregulated above threshold (*Rasl10b*, *Saa3*, *AW112010* and *Timp1*) and the important transcription factor *Myc* (c-Myc) was downregulated. In M6, transcription of four predicted genes (*Gm10600*, *Gm21968*, *Gm10592* and *Gm3893*) previously observed to be downregulated in *S. pn.* infection with serotypes 7F and 4 alone were also downregulated 14 days post IAV infection alone. Importantly, these results indicated resolving IAV infection to sustainably affect the transcriptional profile of AECII in the lung, supporting our rationale to study AECII responses towards *S. pn.* in secondary infection during resolving IAV infection.

The post-IAV AECII transcriptional configuration provided the basis for the AECII response to secondary *S. pn.* infection (Fig. 6B). At 4 h following secondary *S. pn.* infection with serotype 19F, the number of at least 3-fold regulated genes and the resulting partial network remained similar to the IAV-shaped partial network. In line with this, the HIM distances of the IAV (day 14) and the IAV/19F (4 h) conditions were similar (Fig. 6C). In contrast, infection with serotypes 7F and 4 resulted in a rapid enlargement of the AECII partial co-expression network, positioning them much closer to the complete network and setting them clearly apart from the IAV/19F (4 h) partial network in the HIM distance plot. In particular, secondary infection with serotypes 7F and 4 rendered the regulated IFN-response genes in M2 more numerous as compared to IAV/19F infection (4 h). Of note, the comparatively blunted immediate early AECII response to secondary infection with serotype 19F may be linked to the reduced BALF CFU observed in this infection setup (Fig. 1E).

At 18 h post secondary pneumococcal infection, resolving IAV infection clearly enhanced the transcriptional regulation of IFN-response genes in M2 for all serotypes as compared to the respective *S. pn.* infection alone. This effect was most pronounced for 7F and became particularly evident when focusing on the two major interferon-regulatory hub genes *Irf7* and *Stat1* and their network neighbors (Additional file 10). The uniqueness of the AECII response 18 h post IAV/7F infection was also reflected by the HIM distances of the according partial networks, which presented closest to the complete ARACNE network (Fig. 6C). As for *S. pn.* infection alone, serotype 7F also exclusively induced downregulation of most genes attributed to M3 in secondary infection, only now already 4 h post *S. pn.* infection.

Interestingly, most proliferation-associated genes in M4 underwent a major gene regulatory shift 18 h post secondary *S. pn.* infection with any serotype, reversing the proliferative transcriptional configuration of AECII detected 14 days post IAV infection alone. Similarly, many genes in the adjacent module M5 were downregulated as well, again counteracting the IAV-primed induction of this module.

Taken together, ARACNE network inference of the in vivo AECII transcriptional response to different serotypes of *S. pn.* alone or during resolving IAV infection allowed elaborate dissection of serotype-specific transcriptional regulation in AECII. These analyses identified a positive transcriptional synergy between sustained IAV-mediated alterations and *S. pn.* infection, leading to an accelerated and intensified interferon response, particularly in the case of serotype 7F. This finding co-incided with the observed enhanced susceptibility of IAV-infected mice to invasive disease and excess inflammation following secondary infection with *S. pn.* 7F late after IAV infection. In contrast, previous IAV infection had little effect on the transcriptional upregulation of interferon response genes to serotye 4 infection, which *per se* showed the strongest induction as compared to serotypes 7F and 19F. Next to enhanced synergistic AECII responses with respect to IFN-signaling, secondary *S. pn.* infection led to an abrogation of the IAV-associated proliferative transcriptional configuration in AECII irrespective of the pneumococcal serotype.

### Resolving IAV infection significantly alters respiratory IFN production following *S. pn.* infection and the responsiveness of AECII to IFNs

**Fig. 7:**
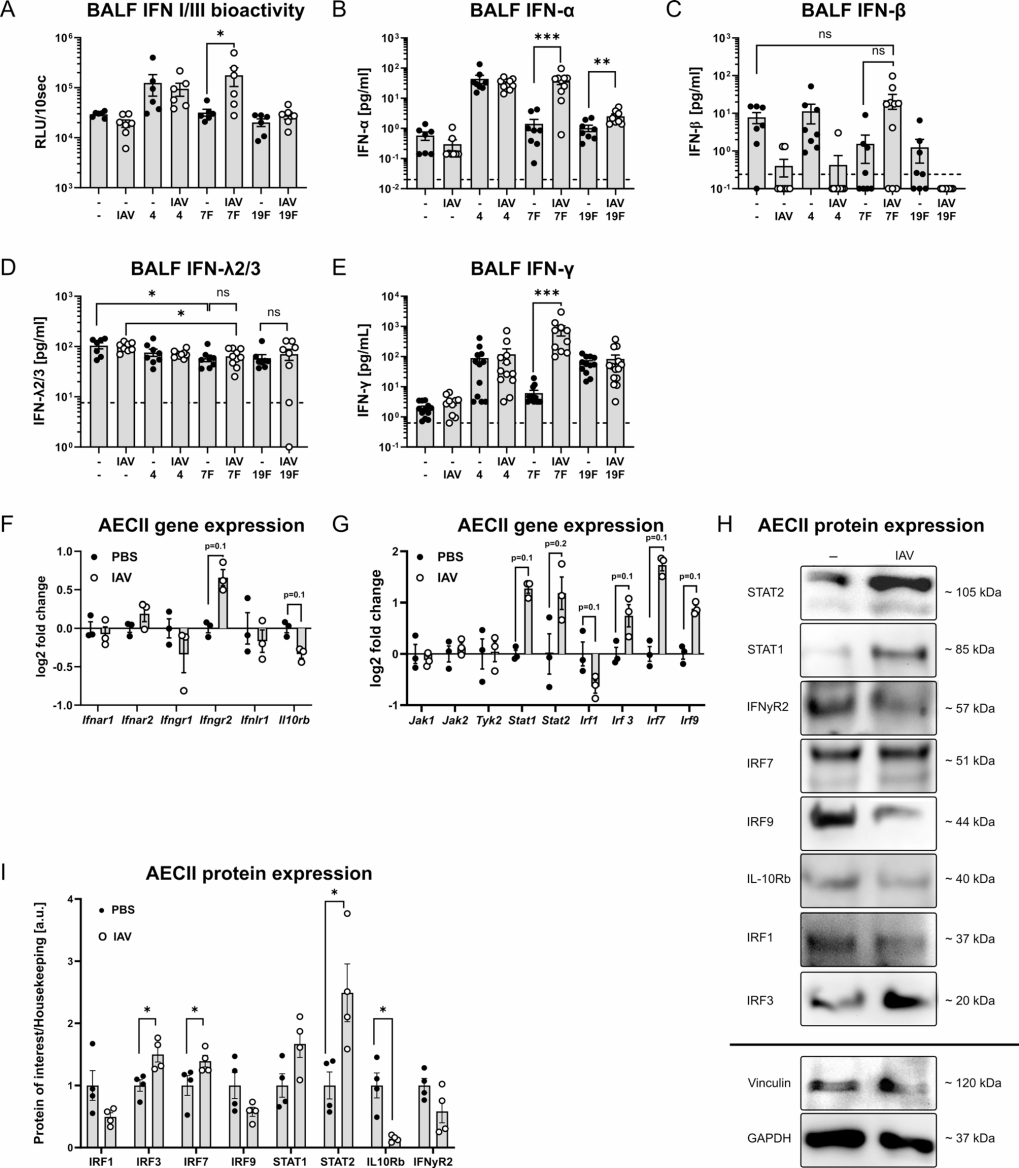
IAV-mediated alterations in airway IFN protein levels and AECII expression of IFN-signaling-related genes. **(A-E)** Mice were intranasally infected with 7.9 TCID_50_ IAV (H1N1, PR/8/34) or treated with PBS. At day 14, IAV-infected and PBS-treated mice were oropharyngeally infected with 10^6^*S. pn.* (serotype 4, 7F or 19F). Levels of bioactive type I/III IFNs **(A)**, IFN-α **(B)**, IFN-β **(C)**, IFN-λ **(D)** and IFN-γ **(E)** were determined in BALF 18 h post pneumococcal infection using an Mx2-luc-reporter assay **(A)**, ELISA **(B**,** C**,** D)** or bead-based multiplex assay **(E)**. Data were obtained from at least two independent experiments. Statistical analyses were performed by two-way ANOVA and Tukey‘s multiple comparisons test, *p* < 0.05, *p* < 0.01, *p* < 0.001. **F-I)** Gene transcription and protein expression in AECII were analyzed by microarray or western blot. AECII from *n* = 3 – 5 mice/experimental group/experiment were pooled. Depicted are individual data and mean ± SEM from at least three independent infection experiments. Statistical analysis was performed by two-tailed Mann-Whitney test, *p* < 0.05. **F)** Relative expression of IFN receptor genes. **G)** Relative expression of IFN signal transducer genes. **H)** Representative blots from AECII protein lysates. **I)** Relative expression of selected IFN signaling proteins, normalized to the expression of the housekeeping proteins vinculin or GAPDH

We assessed, whether the observed changes in the post-influenza AECII response to *S. pn.* and especially in their IFN signaling were associated with an altered abundance of airway IFN in secondary *S. pn.* infections. To this end, we compared levels of bioactive IFN I and III in BALF from mock- as well as IAV-, *S. pn.*- and IAV/*S. pn.*-infected mice using type I and III IFN-sensitive epithelial cells isolated from Mx2-Luc reporter mice [27]. Airway IFN I/III levels in post-IAV (day 14), 7F as well as 19F-infected (18 h) mice were similar to baseline airway IFN levels in controls. Infection with *S. pn.* serotype 4 strain was associated with elevated bioactive airway IFN I/III levels, independent of previous IAV infection. In contrast, previous IAV infection was associated with significantly increased IFN I/III-bioactivity upon infection with the *S. pn.* 7F strain as compared to 7F infection alone (Fig. 7A). Further, we quantified BALF IFN-α, -β, -λ2/3 and -γ. Similar to the results obtained by IFN bioassay, previous IAV infection greatly enhanced IFN-α responses in the airways upon *S. pn.* 7F infection, reaching similar IFN-α levels as *S. pn.* serotype 4 infection (Fig. 7B). Interestingly, we further observed that, as compared to baseline, airway IFN-β levels were reduced 14 days following IAV infection alone and were not significantly elevated following infection with either *S. pn.* serotype 4, 7F or 19F alone. In contrast, secondary infection with *S. pn.* 7F following IAV infection enhanced IFN-β responses as compared to 7F alone, yet without statistical significance (*p* = 0.0733) and only to concentrations that were comparable to baseline levels (Fig. 7C). In contrast to type I IFN production, we only detected minor changes in BALF type III IFN levels. Here, infection with *S. pn.* 7F alone or following IAV infection was associated with a small, but significant decrease of IFN-λ2/3 as compared to baseline or to IAV infection alone, respectively (Fig. 7D). With respect to BALF IFN-γ levels, we observed a strong and significant increase between *S. pn.* infection alone and secondary *S. pn.* infection exclusively for serotype 7F (Fig. 7E). Of note, dedicated interrogation of the microarray data for AECII interferon gene expression revealed expression (in descending strength) of *Ifna5*, *Ifnl3*, *Ifnl2*, *Ifna9*, *Ifna15*, *Ifna6* and *Ifna1* above median level (Additional file 4) which was remarkably stable across all experimental conditions.

We further asked, whether IAV-mediated intrinsic changes in the AECII responsiveness to IFNs contributed to their altered IFN-associated gene transcriptional response in secondary *S. pn.* infection. Thus, we analyzed the transcription and protein expression of molecules involved in IFN detection and signaling in AECII isolated from controls and IAV-infected (day 14) mice. In the gene transcriptional data, we detected increased gene expression of *Ifngr2* at day 14 post IAV, whereas expression of *Il10rb* was decreased (Fig. 7F). Further, the transcription of genes for various intracellular interferon signal transducers (*Stat1*, *Stat2*, *Irf3*, *Irf7*, *Irf9*) was increased in AECII after IAV infection, while only expression of *Irf1* was reduced (Fig. 7G). Except for IFNGR2 and IRF9, we found that transcriptional changes were similarly detected on the protein level in AECII (Fig. 7I, H and Additional file 11). Taken together, 14 days post IAV infection, AECII expressed increased levels of the IFN response transcription factor proteins STAT2, IRF7 and IRF3 as compared to AECII from uninfected controls. In line with core observations of the AECII transcriptome analysis, this cellular configuration potentially contributed to the characteristic presentation of secondary *S. pn.* serotype 7F infection, potentiating the AECII response to type I and II interferons, as detected for this serotype in comparison to serotypes 19F and 4.

### IAV infection induces epigenetic imprinting of AECII MHC-class II expression and IFN responses

**Fig. 8:**
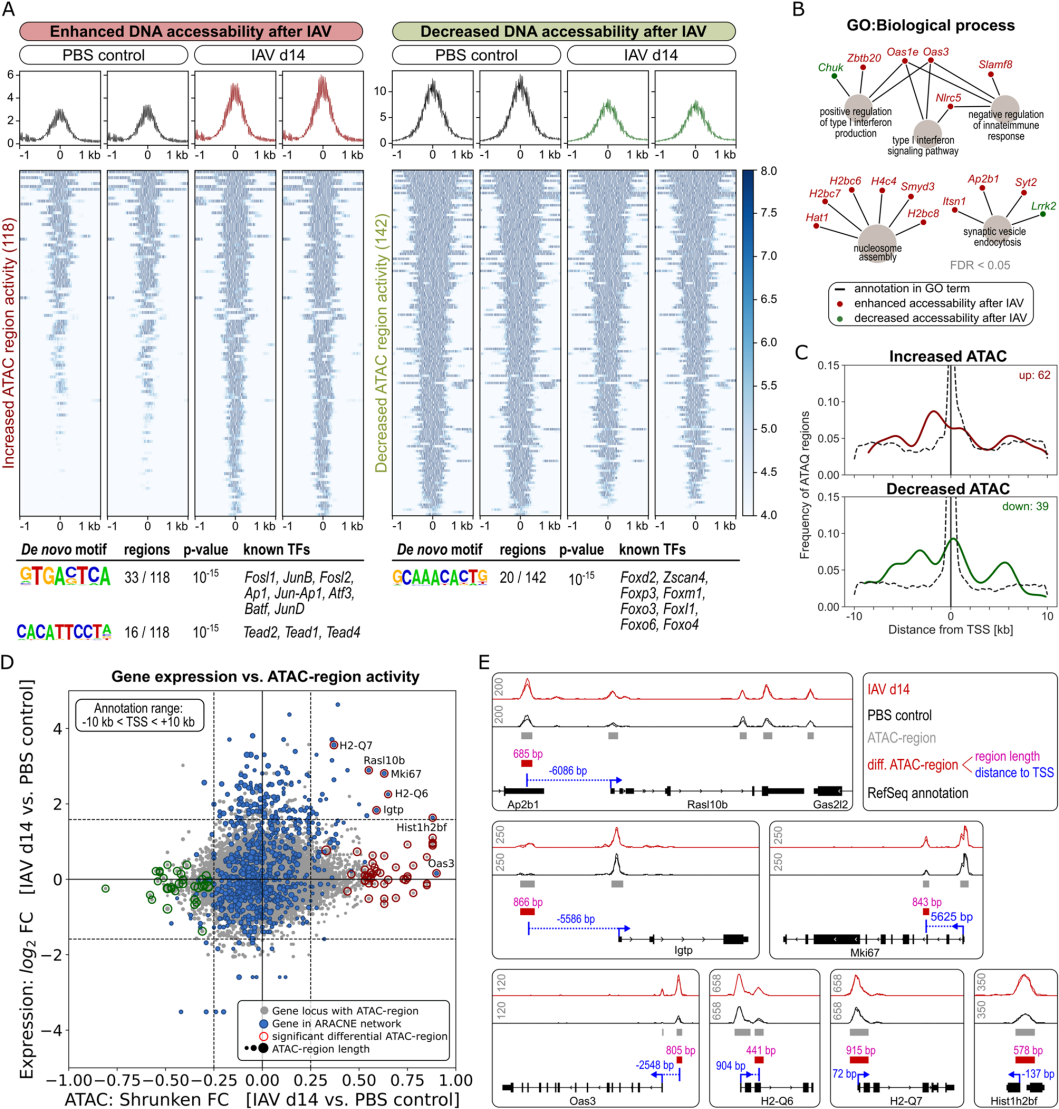
IAV infection induces epigenetic imprinting of MHC-class II and IFN-response genes in AECII. Mice were intranasally infected with 7.9 TCID_50_ IAV (H1N1, PR/8/34) or treated with PBS. After 14 days, AECII were isolated (*n* = 3 – 5 pooled mice per experimental group) in two replicate experiments and chromatin was analyzed by ATAC-seq. DNA regions with differential ATAC state (|shrunken log_2_ FC| > 0.25, FDR < 0.1) comparing IAV day 14 AECII vs. PBS control AECII were identified. **(A)** More (118) and less (142) active ATAC regions were aligned at region centers and normalized ATAC signals within a window of ± 1 kb were clustered and color-coded. Significant results of HOMER analysis for transcription factor binding motifs and known binding TFs are indicated below. **(B)** Gene Ontology (GO) analysis (two-sided hypergeometric test, FDR < 0.05) of genes annotating to differentially active ATAC regions for the GO category “biological process”. **(C)** Histogram showing relative localization of differentially active ATAC regions. Only regions with gene annotations that fall into a window of ± 10 kb around transcription start sites (TSS) were included. Dashed lines: Localization of unthresholded ATAC regions. **(D)** Scatterplot correlating genes with at least one differentially active ATAC region within a window of ± 10 kb of TSS and respective microarray gene expression data. Each grey point represents an annotated ATAC region. Point size represents ATAC region length. Microarray-based log_2_ FC of gene symbols are plotted vs. ATAC-based shrunken log_2_ FC of matching ATAC regions. ATAC regions annotating to gene symbols that are part of the ARACNE network are shown in blue. Significantly less/more active ATAC regions are indicated by green and red circles, respectively. **(E)** Locus representation of genes of the ARACNE network that yielded significant differential activity of ATAC regions. Data represent overlaid ATAC signals from two independent replicates of AECII from PBS controls and resolving IAV infection (day 14). Grey numbers in upper left corners indicate normalized bigwig-data scaling

Based on the sustained IAV-associated transcriptional footprint in AECII, we hypothesized presence of IAV-mediated epigenetic imprinting in these cells. According to this hypothesis, epigenetic imprinting of AECII transcriptional responses could underlie the faster and stronger response of AECII to interferons upon secondary *S. pn.* encounter. To address this hypothesis, we analyzed AECII from IAV-infected (day 14) and control mice by ATAC-seq analysis (Fig. 8). In total, we identified 83,681 ATAC regions (of which 78 % were annotatable) within AECII chromatin (from two independent replicates) over both conditions (Additional file 12). From these, 260 regions showed significant differential ATAC activity (|shrunken log_2_ FC| > 0.25, FDR < 0.1). Of these 260 regions, 118 showed enhanced and 142 regions decreased chromatin accessibility (Fig. 8A). Enhanced regions were systematically shorter (median: 670 bp) than regions with decreased chromatin accessibility (median: 818 bp). *De novo* HOMER motif-enrichment analysis indicated significant overrepresentation of three sequence motifs (Fig. 8A bottom). The main motifs GTGACTCA (33 sites) and CACATTCCTA (16 sites), found within ATAC regions with enhanced accessibility after IAV infection, are known to be compatible with JUN/FOS (AP-1) and TEAD (TEAD1, 2 and 4) transcription factors, respectively. ATAC regions with impaired accessibility related to the family of forkhead-binding-domain-containing transcription factors with the main motif GCAAACACTG (20 sites). Of note, *Foxm1* was one of the forkhead-motif-binding transcription factors also part of the AECII ARACNE network (M4), providing further clues to the consequences of diminished chromatin accessibility of according forkhead-motif sites.

GO (biological process) annotation of the gene loci positioned within a broad annotation window around the identified, differentially accessible regions showed association with type I interferon signaling and production (*Chuk*,* Zbtb20*,* Oas1e*,* Oas3*,* Nlrc5* and *Slamf8*), nucleosome assembly (*Hat1*,* H2bc7*,* H2bc6*,* H4c4*,* Smyd3* and *H2bc8*) and vesicle endocytosis (*Itsn1*,* Ap2b1*,* Syt2* and *Lrrk2*; Fig. 8B). As expected, on the genome-wide scale, the 83,681 ATAC sites in AECII were mainly positioned around transcription start sites (TSS), as shown in Fig. 8C (dashed lines). Constriction of the differential ATAC region annotation window to ± 10 kb around adjacent TSS limited the number of relevant sites to 62 with significantly enhanced ATAC activity and 39 with reduced activity, respectively. Interestingly, ATAC sites with enhanced accessibility were favored to be positioned in proximal promoters up to ‑4 kb upstream of TSS, whereas most sites with decreased accessibility were positioned around TSS (Fig. 8C). To match results from ATAC-seq analysis of AECII 14 days after IAV infection with the ARACNE gene co-expression network, we combined both approaches based on fold-differences over the PBS control. Figure 8D shows a scatterplot that represents shrunken log_2_ ATAC region fold changes of all identified ATAC sites (gray points; significance of region accessibility is indicated by red/green circles) positioned in a window of ± 10 kb around genetic start coordinates of known annotated murine gene loci alongside with the matching log_2_ fold change of transcription in the AECII transcriptional regulation dataset (IAV day 14 vs. control). This approach led to the identification of seven gene candidates (*Hist1h2bf*, *Igtp*, *Mki67*, *Rasl10b*, *H2‑Q6*, *H2-Q7* and *Oas3*) that were part of the ARACNE network and yielded an ATAC region with enhanced accessibility in the vicinity of their locus start point. Transcript expression of all candidates with the exception of *Oas3* was significantly elevated in AECII 14 days post IAV infection over the PBS control. These findings suggested that in AECII, enhanced expression of *Hist1h2bf*, *Igtp*, *Mki67*, *Rasl10b*, *H2‑Q6* and *H2-Q7* following IAV infection relies on an epigenetic regulatory component. Figure 8E shows the locus ATAC states of all these gene candidates. *Igtp* (interferon gamma-induced GTPase) and *Oas3* (2’-5’-oligoadenylate synthetase 3) belong to the interferon response genes in ARACNE module M2 and were both positively correlated with the hub transcription factor *Irf7*. *Ras10lb* (RAS like family 10 member b), a predicted G protein activator, is part of M1 and was one of few genes with intermodular connections within the ARACNE network, connected to *Ifitm3* in M2 and *B2m* in M5. *H2‑Q6* and *H2-Q7* belong to M5 and were not connected to any adjacent transcription factors but were interconnected with other major histocompatibility genes in the same network module. *Mki67* (marker of proliferation Ki-67) in M4 is connected to transcription factors *E2f8* and *Foxm1*, both related to cell proliferation. *Hist1h2bf* (also termed *H2bc7* or H2B clustered histone 7) is also part of M4 and correlated to other histones from the H2b family.

Taken together, ATAC-seq analyses of AECII isolated 14 days post IAV infection identified six ARACNE network genes that were at the same time transcriptionally upregulated and revealed ATAC regions with enhanced chromatin accessibility in their promoter regions. This finding supports epigenetic imprinting of AECII following IAV infection with clear consequences for their transcriptional regulation and response to secondary infection with *S. pn.*

## Discussion

The epithelial barrier, its breaches and interactions with environmental cues are central to a variety of human diseases [58]. We have previously described rapidly induced, strong transcriptional regulation in AECII during acute IAV infection and at the same time have observed prolonged susceptibility towards *S. pn.* following IAV infection in an *S. pn.* serotype-specific manner [12, 19]. Prompted by these observations, we hypothesized that (i) AECII serotype-specifically respond to *S. pn.* infection in vivo and that (ii) IAV infection affects this response, contributing to the progression of secondary pneumococcal infection. Indeed, we detected robust, serotype-dependent regulation of gene transcription in AECII in response to *S. pn.* alone. While IFNs I and III are classically associated with viral infections, they also play a role in anti-bacterial defense mechanisms [13, 59, 60]. In the AECII transcriptional response to *S. pn.* infection, the interferon hub transcription factors *Stat1* and *Irf7* were highly induced in response to all three serotypes. Serotype 4 further stood out with an exceptionally strong induction of the genes within the respective ARACNE module M2. Linking this transcriptional response to respiratory mediator levels, BALF IFN-α and -β levels were highest in response to serotype 4 infection as compared to 19F and 7F. As transcription of genes for IFNs was not particularly regulated in AECII, this finding underlines the role of the inflammatory milieu in shaping the AECII response to infection.

Resolving IAV infection increased invasiveness of serotypes 4 and 7F as compared to *S. pn.* infection alone. This contrasted significantly improved bacterial clearance of serotype 19F in secondary infection as compared to 19F infection alone and demonstrated increased invasiveness of *S. pn.* following IAV infection not to display a general, exclusively host-dependent phenomenon. With respect to the mechanisms shaping serotype-specific inflammation and AECII responses, the pneumococcal capsule represents a strong candidate. It is the key determinant of individual pneumococcal serotypes, a prerequisite for invasiveness and thereby a major virulence factor [61]. Only encapsulated strains can establish disease, while at the same time, production of capsule components involves energetically costly mechanisms [62]. Invasive serotypes were shown to produce thinner, energetically more costly capsules as compared to colonizing serotypes. By exchanging serotype-specific capsule operons, the according serotype’s growth phenotype in nutrient-restricted conditions is also transferrable [62]. In this context, it has been assumed that bacterial nutrient availability in the airways is affected by IAV infection and contributes to enhanced susceptibility to secondary bacterial infection following IAV infection [63]. Capillary leakage during IAV infection has indeed been shown to provide nutrients and antioxidants supporting *S. pn.* outgrowth [7]. In this context, we hypothesize that several host-mediated as well as bacterial-intrinsic factors potentially contribute to the phenomenon of improved clearance of the 19F strain in post-influenza lungs. Given the fact that significantly reduced CFU in 19F superinfected airways were already detected at 4 h post infection (compared to 19F CFU in IAV-naive lungs at this time point), we assume that the post-IAV airway microenvironment immediately impeded microbial attachment and growth and/or facilitated innate immune-mediated clearance of bacteria in the lungs. It was previously demonstrated that differences in capsule composition affect the deposition of complement factors (opsonization) on the bacterial surface, which in turn contributes to strain-specific differences in bacterial immune evasion [64, 65]. Interestingly, lung epithelial cells, including AECII ([66] and this study), constitutively produce a wide array of complement proteins which are secreted into the bronchoalveolar space. In this context, we speculate that deposition of complement on the 19F capsule might be more efficient in comparison to the 4 and 7F strains, respectively. Given the fact that post-influenza airways contain increased blood-derived constituents (Fig. 1B), we assume that this might provide an additional source of complement proteins. Besides that, the pneumococcal capsule is also the target of natural secretory IgA (sIgA) and IgM (sIgM). Deposition of these polyspecific immunoglobulins vitally contributes to immune exclusion of pathogenic microorganisms and constitutes a non-redundant mechanism of mucosal innate immunity [67]. Importantly, sIgA and sIgM are transported onto the mucosal surface via the polymeric immunoglobulin receptor (PIGR), which is expressed by airway epithelial cells. In this context, our analyses revealed significant induction of *Pigr* in post-flu AECII (see e.g. Additional file 5). We and others have previously reported that increased airway epithelial PIGR protein levels during lung inflammation are associated with increased sIg levels [68, 69] and increased sIg deposition on pneumococci [68]. Although we are not aware of studies dissecting strain-specific susceptibility of pneumococci to sIg-deposition, we assume that pneumococcal capsule composition directly determines the efficacy of sIg attachment (similar to susceptibility to complement deposition). Altogether, IAV-primed PIGR-mediated sIg transport onto the mucosal surface is a plausible mechanism by which AECII could modulate (strain-specific) immunity towards *S.pn.*

Next to potential differences in susceptibility to innate immune mediated factors between highly invasive (4 and 7F) versus the non-invasive 19F strain, it is conceivable that bacterial adaptation to the inflamed respiratory tract is less efficient in the 19F strain. Sender et al. [7] described pneumococcal adaptation to an oxidized microenvironment in IAV/pneumococcal co-infected lungs to be crucial for bacterial growth and evasion of complement-mediated opsono-phagocytosis. Importantly, their study also describes strain-specific differences in the ability of pneumococci to cope with oxidative stress factors (which are present in the inflamed lower airways), thus highlighting the concept of bacterial adaptation as a major factor determining the pathogenesis of IAV-associated pneumococcal superinfections. In this context, comparative analyses of the bacterial transcriptome and/or translatome would provide critical insights into differences in host/bacterial interplay that might underlie bacterial-strain specific susceptibility to pneumococci in the post-influenza lung. According to these considerations, the serotype-dependent AECII transcriptional responses and their modulation in secondary infection following IAV infection detected in our study most likely to a large extent depend on the pneumococcal surface/capsule composition, associated nutrient consumption and availability, its interaction with the specific immune micromilieu and the induced host response. Here, an additional possible pathway could be the link between pneumococcal sugar transporter expression, neutrophil recruitment and tissue tropism [70]. In this context, particularly strong AECII transcriptional regulation of the neutrophil-attractant CXCL5 in IAV/7F infection as opposed to secondary infection with the other bacterial strains is interesting, as it has recently been described to be produced by AECII and induce barrier permeability in lung injury and pneumococcal infection [71]. Further, particularly nutrient availability and host receptor expression and responsiveness can be affected by IAV infection. While future studies will have to identify the exact mechanisms, bacterial and host factors as well as effector cells that are involved, our study clearly shows AECII to participate in the serotype-specific manifestation of primary and post-influenza pneumococcal infection.

The AECII response to secondary infection with a particular serotype qualitatively and quantitatively never equaled the response to infection with that serotype alone, despite serotype-specific manifestations. This suggests the concurrent presence of a general IAV-associated modulation of anti-pneumococcal AECII responses, independent of the *S. pn.* serotype (or in fact, possibly even the bacterial pathogen) encountered. Generally, we detected a strongly intensified transcriptional regulation of IFN-response genes in AECII following secondary *S. pn.* infection, which was most prominent in secondary 7F infection as compared to 7F infection alone. At the same time, we detected increased IFN-ɑ and IFN-β protein levels in BALF in IAV/7F infection as compared to 7F infection alone. While the increase in IFN-ɑ was statistically significant, the increase in IFN-β was not, despite a clearly elevated mean concentration. While the lack of statistical significance could have risen from biological variability in the infection/response in combination with the sample size, the overall clearly increased BALF IFN I levels (IFN-ɑ and IFN-β) were well in line with significantly increased IFN I/III bioactivity detected in BALF samples from IAV/7F infection as compared to 7F infection alone. Of note, this increase in IFN I/III bioactivity was detected despite significantly reduced IFN-λ protein levels in IAV/7F infection and 7F infection alone as compared to baseline. This reduction was small and there were no alterations in BALF IFN-λ between IAV/7F and 7F infection alone. Therefore, we believe it had limited relevance for the increase in IFN I/III bioactivity detected in IAV/7F infection as compared to 7F infection alone. While there is a strong overlap in ISG induction between IFN-ɑ/-β and IFN-λ, there are differences in the kinetics and concomitant immunopathology [72, 73]. At this point however, we cannot draw conclusions from our data with respect to functional consequences of significantly reduced IFN-λ in IAV/7F and 7F infections for immune function and inflammation in that setting, and future studies would be needed, e.g. employing (cell-type specific) receptor-deficient mice.

Alterations in the secondary response to *S. pn.* as compared to infection with *S. pn.* alone were detected on top of sustained changes detected in AECII 14 days post IAV infection alone. Importantly, IAV-mediated changes in AECII were evident both on the gene transcriptional as well as on the epigenetic level, supporting the concept of inflammatory imprinting or peripheral trained immunity induced in AECII. As AECII act as progenitor cells to constantly replenish AECI pneumocytes [14], our findings may help to understand long-term inflammatory imprinting of respiratory mucosa pneumocytes other than AECII. Particularly via the propagation of epigenetic changes from infection-experienced progenitor AECII to progeny AECI, acute infection could broadly and sustainably affect the lung epithelium beyond AECII. Such mechanisms will need to be addressed in future studies and could well have implications for novel approaches of pharmacological expansion of AECII for the stimulation of regenerative repair [74].

Given that 14 days post IAV infection, BALF levels of IFN-α, IFN-β and IFN-γ were comparable to or even lower than in the PBS control group, it may surprise that AECII anyhow continued to express a robust set of IFN-response genes, accompanied by significantly elevated protein levels of IRF1 and STAT2. Although we did not assess the phosphorylation status of STAT-proteins, this nevertheless may point at an altered IFN detection threshold due to sensitized IFN signaling cascades, or to a long-lasting transcriptional IFN-stimulated gene (ISG) locus memory that, to some extent, is independent of classical ISG induction mechanisms, potentially via phosho-STAT protein complexes. Keeping in mind the accelerated and intensified transcriptional AECII response to interferons upon secondary *S. pn.* infection, persistence of IAV-primed ISG expression in AECII is worth considering. Indeed, there is evidence for IFN-independent and -dependent roles of non-phosphorylated STAT1 and STAT2 for basal and long-term ISG expression [75], providing a concept for non-canonical ISG expression. Of note, the described IAV-mediated alterations were detected at a time-point when viral clearance was completed, thereby excluding IAV remnants as a trigger for late IFN-induction [12].

Using an imiquimod model of skin inflammation and the analysis of epidermal stem cells, deep mechanistic insight into inflammatory memory on the cellular level, that might also apply to explain persistent ISG expression in post-IAV AECII, has been described [76]. In their model, Larsen *et al.* demonstrate that cell-type and stimulus-specific transcription factors in conjunction with common stress-response transcription factors FOS-JUN (also known as AP-1) set genomic memory domains. The associated gene loci later take part in a transcriptional inflammatory memory recall. According to this concept, JUN eventually remains bound to the initially generated genomic memory domains and together with further homeostatic transcription factors is responsible for an accessible chromatin state of memory domains. In case of a secondary inflammatory stimulus, stress-induced FOS is re-recruited independently of the initially required stimulus-specific transcription factors and ultimately induces fast and efficient transcription of genes associated to the memory domains [76]. As ATAC-seq analysis of AECII 14 days post IAV infection identified 33 genomic regions with enhanced chromatin accessibility that contain binding motifs for JUN/AP-1 transcription factors, the model suggested by Larsen *et al.* may also apply to AECII. This provides a further mechanistic explanation for the persistent ISG expression that is likely to accelerate and enhance the AECII ISG response in secondary *S. pn.* infection. In line with this, ARACNE network module M1 contains five genes (*Ccl19*, *Dnaja1*, *Dusp19*, *Fzd2* and *Ptpn1*) that are GO-annotated to regulate JUN kinase activity, with JUN phosphorylation being biochemically important for JUN activation. Further studies will be required to conclusively link chromatin accessibility of ATAC-identified AP-1 sites in IAV-imprinted AECII to the expression of the observed set of persistently expressed IFN-response genes.

Several studies have previously shown roles for IFN I signaling in secondary bacterial complications following IAV infection, specifically attributed to functional alterations in γδ-T cells or alveolar macrophages [9, 77–79]. With respect to AECII, disruption of lung epithelial repair during recovery from IAV infection through type I and III IFNs displays one plausible mechanism linking virus-associated IFN signaling to enhanced susceptibility to secondary *S. pn.* infection [13]. In contrast, IFN I signaling in AECII has been shown to protect mice from *S. pn.* by promoting AECII survival [59]. Further, IFN I has been shown to protect against invasive disease by inhibiting pneumococcal transmigration across the lung [80]. Therefore, dissection of virus- and pneumococci-induced IFN responses and their possibly divergent consequences will be important. In the light of the broad induction of ISG in AECII, the bacterial strain-dependent differences in respiratory IFN levels and the potentially divergent effects of IFN signaling in viral and bacterial infections, it is difficult to mechanistically assign the observed effects, particularly with respect to 7F and IAV/7F infection, to single ISG candidates. Nevertheless, there are candidates such as *ISG15*. It is a central ISG broadly acting in host immune reactions [81] and was most prominently upregulated in IAV/7F infection as compared to IAV/19F and IAV/T4 infections. *ISG15* has been shown to be induced in respiratory epithelial cells by *S. pn.* D39 [82] and a strain-dependent uptake and intracellular degradation of *S. pn.* and thereby *ISG15* induction resulting in serotype-specific manifestation of the infection is feasible. However, further experimental work also with respect to epithelial uptake of the different *S. pn.* strains will be needed for clarification. Further, the IFN I inducible gene *Cxcl13* stood out in IAV/7F infection as highly upregulated on the AECII gene transcriptional as well as BALF protein level. However, while it is associated with B cell responses and ectopic germinal center formation in IAV infection [83], to our knowledge little is known about its role in acute pneumococcal infection to date.

Another *S. pn.* serotype-independent phenomenon observed upon secondary infection with all three serotypes was the swift blunting of genes in the AECII proliferation-related network module M4. This module’s transcriptional activity supposedly is a consequence of post-IAV tissue repair processes. Interestingly, IFN-α, IFN-β and particularly IFN-λ were demonstrated to impair AECII proliferation in regenerating mouse lungs post IAV infection, with implications for the severity of *S. pn.* secondary infection. Consequently, IFNLR-knockout mice show improved post-IAV AECII proliferation and are less susceptible to *S. pn.* secondary infection [13]. This is in line with another previous report of significant loss of epithelial cell re-proliferation and repair responses in secondary pneumococcal infection two days post IAV infection [84]. Also, in a recent report of longitudinal lung tissue transcriptome analyses in secondary *S. pn.* infection that was performed five days following IAV infection, the top downregulated differentially expressed genes in IAV/*S. pn.* infection as compared to single infections were involved in the regulation of epithelial cell proliferation. Also, ISG (including *Irf7*) were switched from positive to negative correlations with the host resistance state upon secondary pneumococcal infection in that study [85]. In our study, given the persistent AECII ISG expression post IAV infection, the rapid shutdown of the AECII mitotic transcriptional configuration may be a direct consequence of an increased AECII responsiveness to IFNs during secondary *S. pn.* infection.

So far, AECII are not considered major players in anti-pneumococcal immune responses. Clearance of colonization and infection with *S. pn.* depend on classical immune cells such as monocytes and macrophages, supported by CD4^+^ T cells [86, 87]. Further, the complement system and recruited neutrophils contribute to the uptake and killing of *S. pn.*, altogether associated with intense inflammation and cytotoxicity [87, 88]. Humoral adaptive immunity is induced and while antibodies are not able to clear established colonization, they are essential for effective vaccination [89, 90]. Our study clearly shows that AECII should be taken into account when aiming at a full understanding of seroytype-independent and -dependent interactions between *S. pn.* and its host in primary and post-influenza infection.

## Conclusions

Our study underlines that enhanced susceptibility to invasive *S. pn.* infection can be sustained beyond viral clearance and is pneumococcal serotype-specific. We extend the current knowledge, showing that AECII as resident cells of the respiratory mucosa sustainably retain an IAV-associated transcriptional configuration encompassing epigenetic changes. These sustained IAV-mediated changes have implications for the AECII response in secondary pneumococcal infection, characterized by a faster and more vigorous response to IFNs and reprogramming of proliferation. While future studies will have to decipher the details of how these AECII-intrinsic processes relate to the *S. pn.* serotype-specific manifestation of secondary pneumococcal infection late after IAV infection and thereby contribute to the synergism between influenza viruses and secondary bacterial pathogens, the results of our study clearly show that AECII need to be considered as potent contributors to inflammatory tissue imprinting in the respiratory tract following IAV infection.

## Electronic supplementary material

Below is the link to the electronic supplementary material.

Additional file 1: Applied FACS gating strategy for AECII isolation and AECII purity analyses.

Additional file 2: Viral burden during acute IAV pneumonia.

Additional file 3: Airway cytokine and chemokine levels in S. pn. and IAV/S. pn. infection.

Additional file 4: Expression of interferon genes in AECII.

Additional file 5: Complete microarray expression dataset.

Additional file 6: Node and edge list of ARACNE network.

Additional file 7: Module assignment of ARACNE network genes.

Additional file 8: Differential AECII ARACNE gene co-expression partial networks.

Additional file 9: Clustering coefficients of ARACNE network nodes.

Additional file 10: Clustered node neighbor expression heatmaps of interferon-hub genes Irf7 and Stat1.

Additional file 11: Western blot quantification of IFN-signaling-related proteins in AECII.

Additional file 12: Identified ATAC regions.

Additional file 13: Additional methods: Viral burden quantification by quantitative real-time RT-PCR.

## Data Availability

The datasets generated and/or analysed during the current study are available in the Gene Expression Omnibus (GEO) repository under reference IDs GSE225343 and GSE225498. Python scripts for the analysis and visualization of transcriptomic data and network representation are available from the corresponding author upon reasonable request.

## Abbreviations

19F: Streptococcus pneumoniae serotype 19F
4: Streptococcus pneumoniae serotype 4
7F: Streptococcus pneumoniae serotype 7F
AECII: Type II alveolar epithelial cells
ARACNE: Algorithm for the Reconstruction of Accurate Cellular Networks
ATAC: Assay for transposase-accessible chromatin
BALF: Bronchoalveolar lavage fluid
DC: Dendritic cells
FC: Fold change
GEO: Gene Expression Omnibus
GO: Gene ontology
H: Hamming distance
HIM: Hamming-Ipsen-Mikhailov
i.n.: Intranasal
i.p.: Intraperitoneal
IAV: Influenza A virus
IFN: Interferon
IM: Ipsen-Mikhailov
IPD: Invasive pneumococcal disease
ISG: Interferon stimulated gene
MI: Mutual information
PAMPs: Pathogen-associated molecular patterns
RLU: Relative light units
*S. pn.*: *Streptococcus pneumoniae*
SI: Signal intensity
SPF: Specific-pathogen-free
TCID_50_: 50% tissue culture infectious dose
TSS: Transcription start sites
