# Supplementary material for: Epigenetic changes and serotype-specific responses of alveolar type II epithelial cells to Streptococcus pneumoniae in resolving influenza A virus infection
Source: Cell Commun Signal. 2025 Jun 12;23:278. doi: 10.1186/s12964-025-02284-y (PMC12164077; doi:10.1186/s12964-025-02284-y)
Supplement: Supplementary file 1 — Additional file 1: Applied FACS gating strategy for AECII isolation and AECII purity analyses. [file 12964_2025_2284_MOESM1_ESM.pdf]

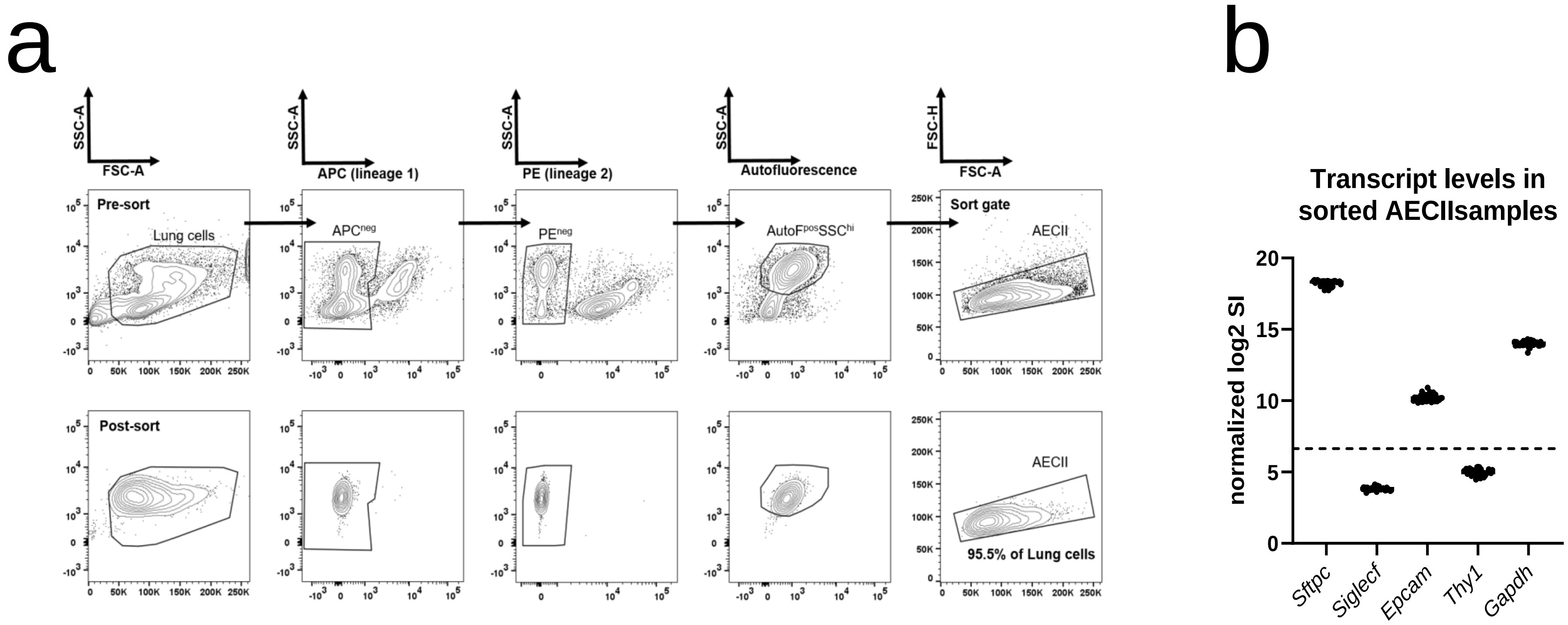

Additional file 1: Applied FACS gating strategy for AECII isolation and AECII purity analyses. a) Lung single cell suspensions were stained with APC- and PE-coupled antibodies against lineage antigens to exclude leukocytes, endothelial cells and platelets. Single AECII (defined as lineage^{neg}, autofluorescence^{pos} and side-scatter^{hi} were sorted (upper panel). Flow-based re-analyses of sorted AECII indicates >95% purity (lower panel). b) RNA from sorted AECII was analyzed by Microarray. Levels of indicated transcripts are expressed as normalized log2 signal intensities (SI). Individual results from each microarray sample (n=42 in total) are depcited (dots), bars show mean transcript levels.
