# Supplementary material for: Epigenetic changes and serotype-specific responses of alveolar type II epithelial cells to Streptococcus pneumoniae in resolving influenza A virus infection
Source: Cell Commun Signal. 2025 Jun 12;23:278. doi: 10.1186/s12964-025-02284-y (PMC12164077; doi:10.1186/s12964-025-02284-y)

Additional file 2: Viral burden during acute IAV pneumonia. Mice were intranasally infected with 7.9 $TCID_{50}$ IAV (H1N1, PR/8/34) or treated with PBS. At day 7 post infection/treament viral nucleoprotein (NP) transcripts in lung tissue were quantified by qPCR. Mean (bars) and individual values from one experiment are depicted. The dashed line indicates the detection limit. Statistical analysis was performed by two-tailed Mann-Whitney test (F,G,H), *p<0.05.

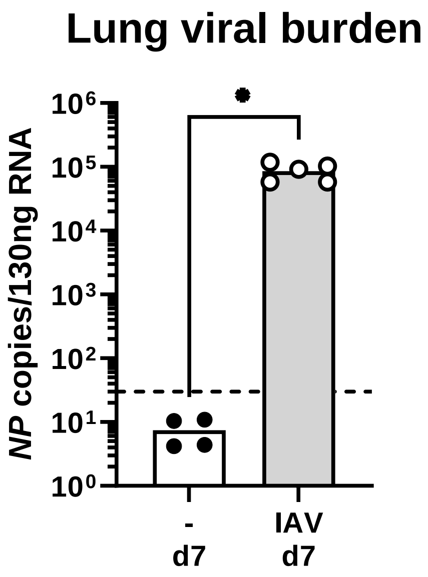

Supplement: Supplementary file 2 — Additional file 2: Viral burden during acute IAV pneumonia. [file 12964_2025_2284_MOESM2_ESM.pdf]
