# Supplementary material for: Epigenetic changes and serotype-specific responses of alveolar type II epithelial cells to Streptococcus pneumoniae in resolving influenza A virus infection
Source: Cell Commun Signal. 2025 Jun 12;23:278. doi: 10.1186/s12964-025-02284-y (PMC12164077; doi:10.1186/s12964-025-02284-y)

Additional file 3: Airway cytokine and chemokine levels in *S.pn.* and IAV/*S.pn.* infection. Mice were intranasally infected with 7.9 $TCID_{50}$ IAV (H1N1, PR/8/34 strain) or PBS. 14 days post primary treatment IAV- and PBS-treated mice were oropharyngeally infected with 10^6 *S.pn.* (serotype 4, 7F or 19F). Cytokine/chemokine concentrations in bronchoalveolar lavage fluid (BALF) were assessed at 4 h or 18 h post bacterial infection by multiplex bead-based immunoassay. Data were compiled from 1 - 3 independent experiments with n = 2 - 7 mice/group/experiment. **a)** Z-scores of mean cytokine/chemokine concentrations of each experimental group were calculated. IAV/*S.pn.* conditions were compared with the according *S.pn.* only conditions and IAV only condtion was compared to PBS control with a two-sided Mann-Whitney-U test. * $p < 0.05$. **b)** Violin plots of baselevel concentration in BAL from mice in the PBS control group and 14 day post IAV infection. Statistical significance indicators were taken over from the z-score analysis in a).

a

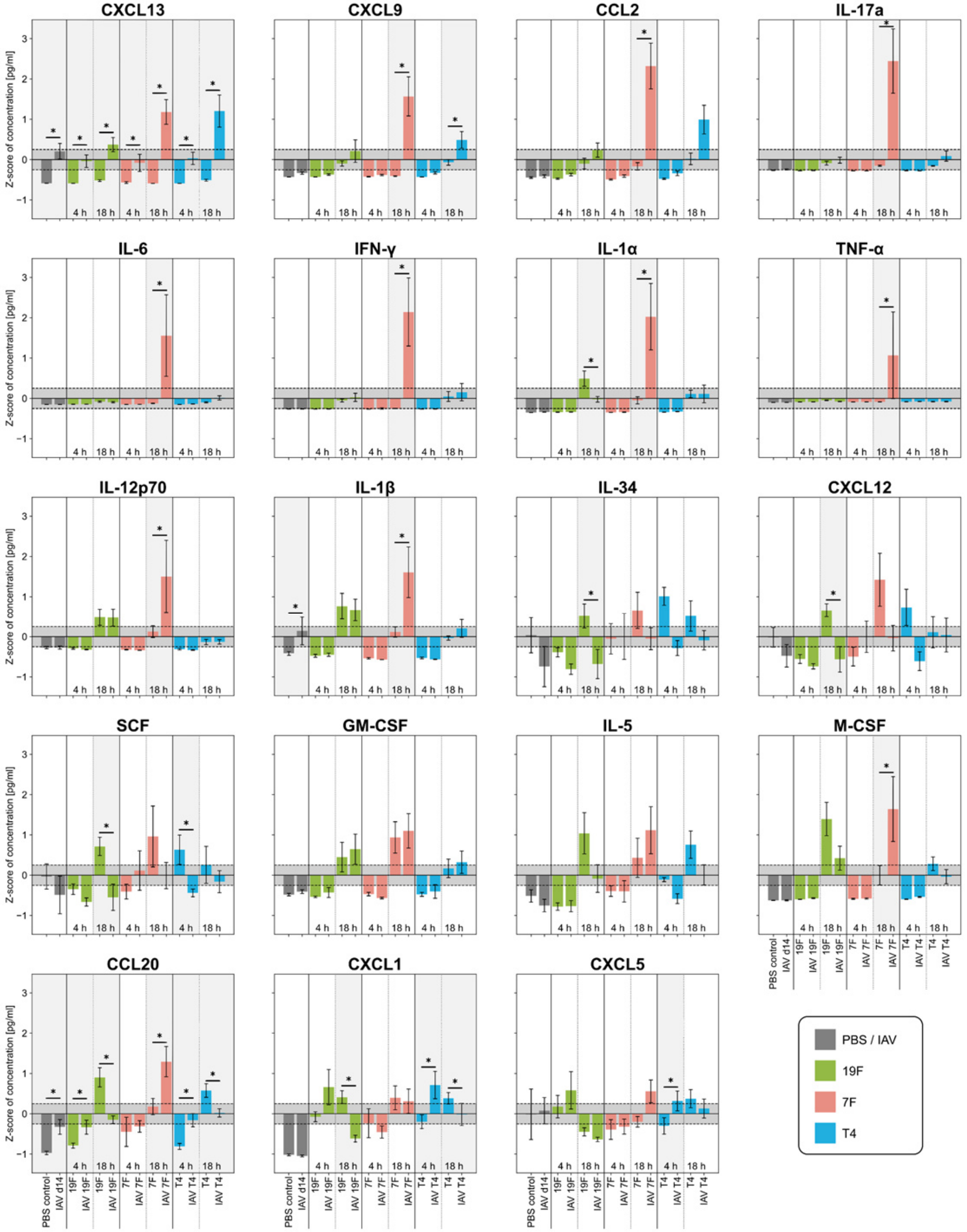

Additional file 3: Airway cytokine and chemokine levels in *S.pn.* and IAV/*S.pn.* infection. Mice were intranasally infected with 7.9 TCID$_{50}$ IAV (H1N1, PR/8/34 strain) or PBS. 14 days post primary treatment IAV- and PBS-treated mice were oropharyngeally infected with 10^6 *S.pn.* (serotype 4, 7F or 19F). Cytokine/chemokine concentrations in bronchoalveolar lavage fluid (BALF) were assessed at 4 h or 18 h post bacterial infection by multiplex bead-based immunoassay. Data were compiled from 1 - 3 independent experiments with n = 2 - 7 mice/group/experiment. **a)** Z-scores of mean cytokine/chemokine concentrations of each experimental group were calculated. IAV/*S.pn.* conditions were compared with the according *S.pn.* only conditions and IAV only condtion was compared to PBS control with a two-sided Mann-Whitney-U test. * $p < 0.05$. **b)** Violin plots of baselevel concentration in BAL from mice in the PBS control group and 14 day post IAV infection. Statistical significance indicators were taken over from the z-score analysis in a).

b

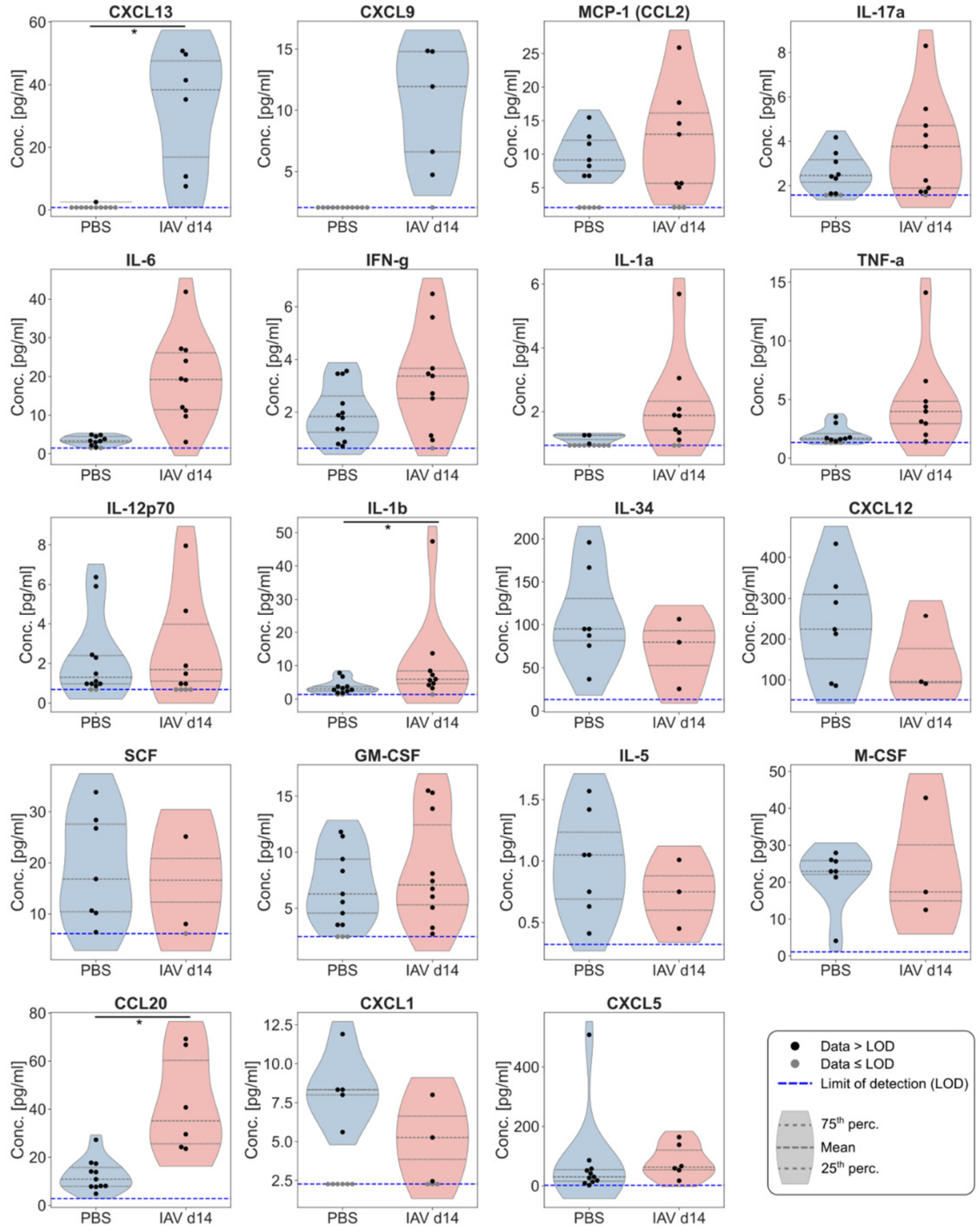

Supplement: Supplementary file 3 — Additional file 3: Airway cytokine and chemokine levels in S. pn. and IAV/S. pn. infection. [file 12964_2025_2284_MOESM3_ESM.pdf]
