# Supplementary material for: Epigenetic changes and serotype-specific responses of alveolar type II epithelial cells to Streptococcus pneumoniae in resolving influenza A virus infection
Source: Cell Commun Signal. 2025 Jun 12;23:278. doi: 10.1186/s12964-025-02284-y (PMC12164077; doi:10.1186/s12964-025-02284-y)

Additional file 4: Expression of interferon genes in AECII. A) Z-scores of normalized $\log_2$ signal intensities (SI) of microarray interferon probesets. Z-scores were calculated only for shown probes per column. **B)** Average microarray SI distribution. Left: relative SI density. Right: Cumulative density. Average SI of interferon microarray probesets are indicated by black lines on the x-axis. Interferons with SI > median are labeled with gene symbols. Median, 25th (q = 0.25) and 75th (q = 0.75) are indicated by blue lines.

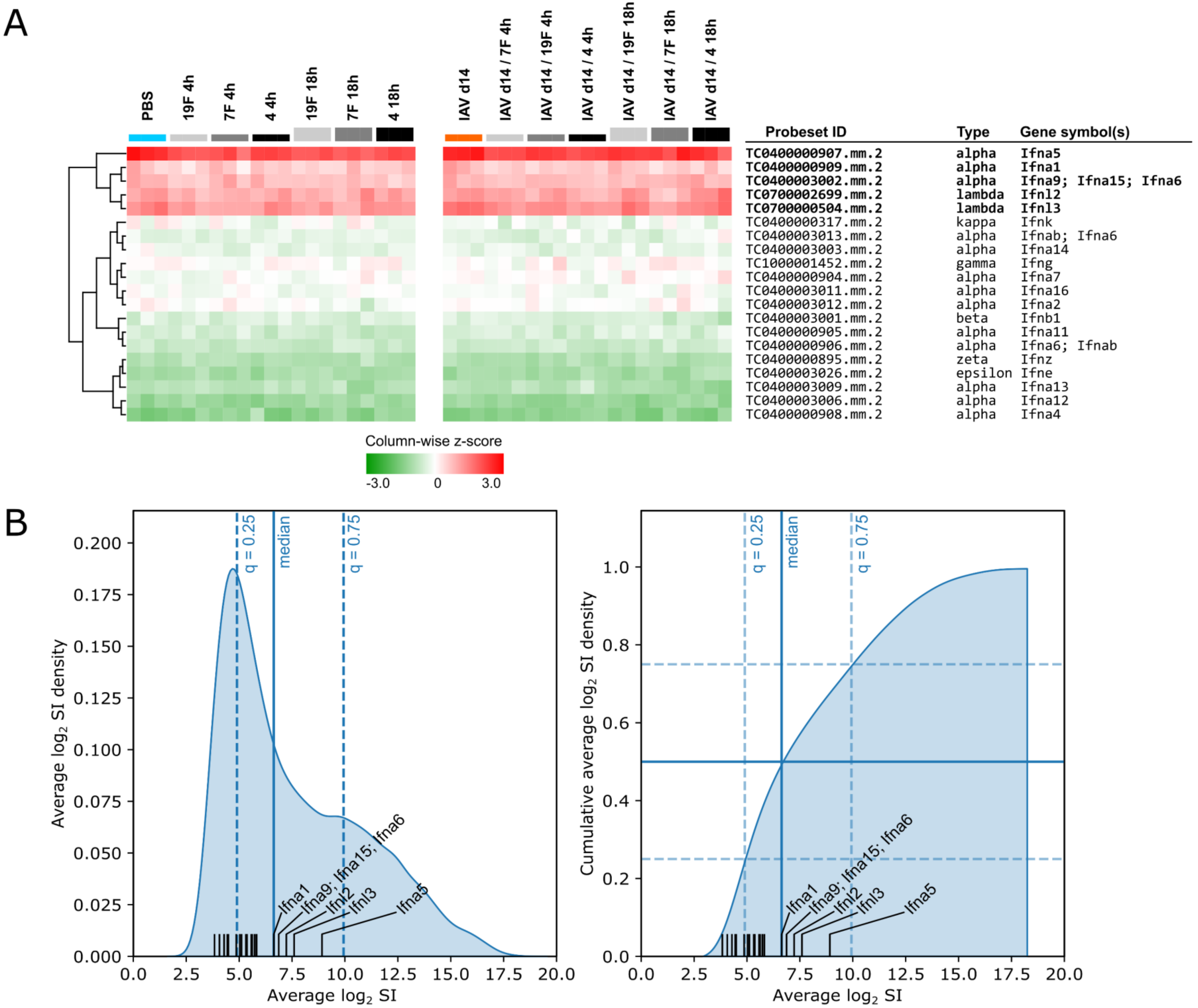

Supplement: Supplementary file 4 — Additional file 4: Expression of interferon genes in AECII. [file 12964_2025_2284_MOESM4_ESM.pdf]
