# Supplementary material for: Epigenetic changes and serotype-specific responses of alveolar type II epithelial cells to Streptococcus pneumoniae in resolving influenza A virus infection
Source: Cell Commun Signal. 2025 Jun 12;23:278. doi: 10.1186/s12964-025-02284-y (PMC12164077; doi:10.1186/s12964-025-02284-y)

Additional file 7: Module assignment of ARACNE network genes. Module assignment of individual genes to ARACNE network modules M1 - M9. Dashed lines indicate module outlines. Nodes are labeled with gene symbols.

Module assignment of ARACNE network genes

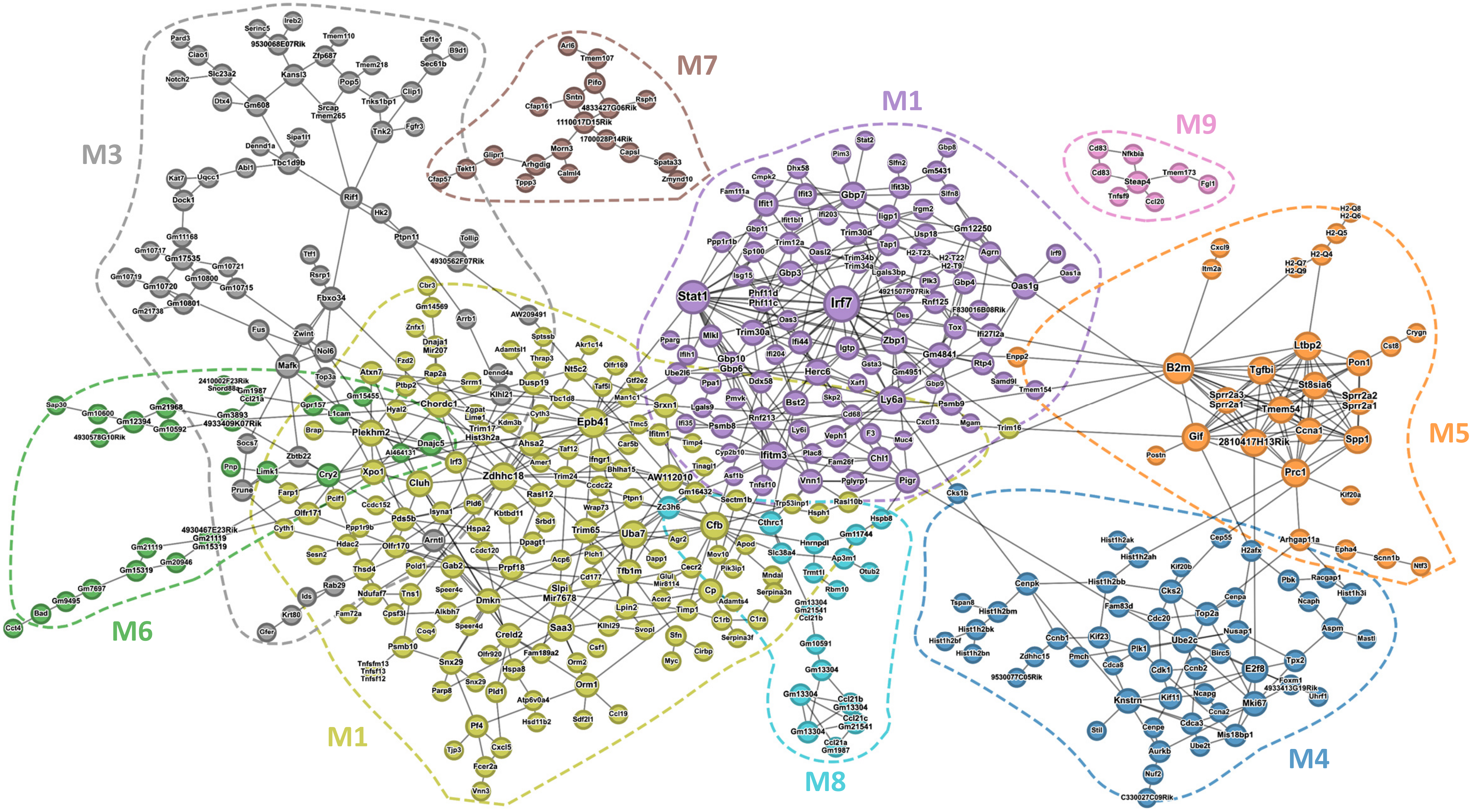

Supplement: Supplementary file 7 — Additional file 7: Module assignment of ARACNE network genes. [file 12964_2025_2284_MOESM7_ESM.pdf]
