# Supplementary material for: Epigenetic changes and serotype-specific responses of alveolar type II epithelial cells to Streptococcus pneumoniae in resolving influenza A virus infection
Source: Cell Commun Signal. 2025 Jun 12;23:278. doi: 10.1186/s12964-025-02284-y (PMC12164077; doi:10.1186/s12964-025-02284-y)

Additional file 8: Differential AECII ARACNE gene co-expression partial networks. Partial networks for genes with |FC| > 3 and their associated edges were calculated for all 13 infection conditions, stated in the upper right corner. Nodes are color-coded according to $\log_2$ FC. Remaining nodes and edges are greyed-out. Node size indicates node connectivity. Colored dashed lines indicate outlines of network modules. Nodes are labeled with gene symbols.

Serotype 19F 4 h vs. PBS control

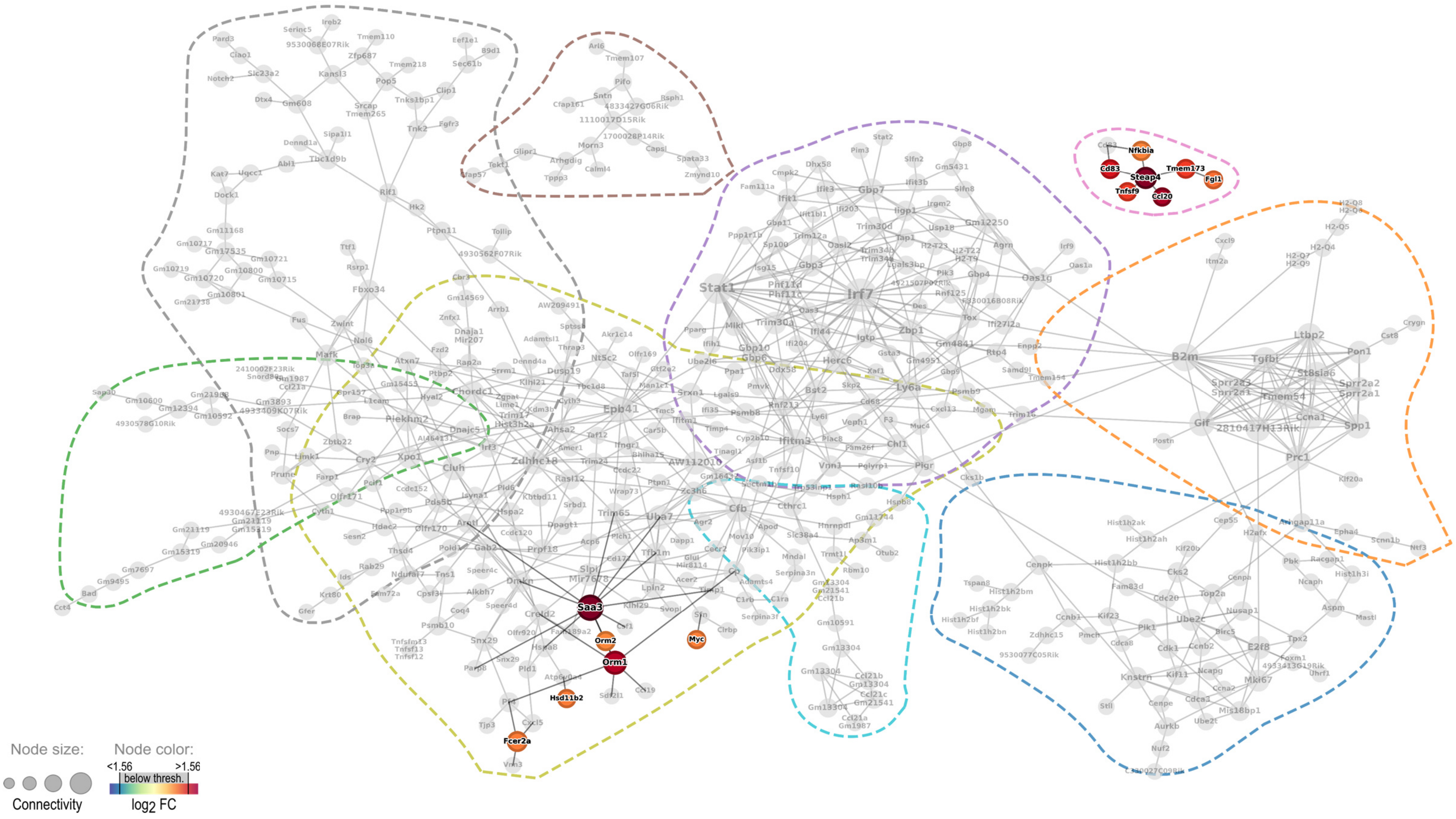

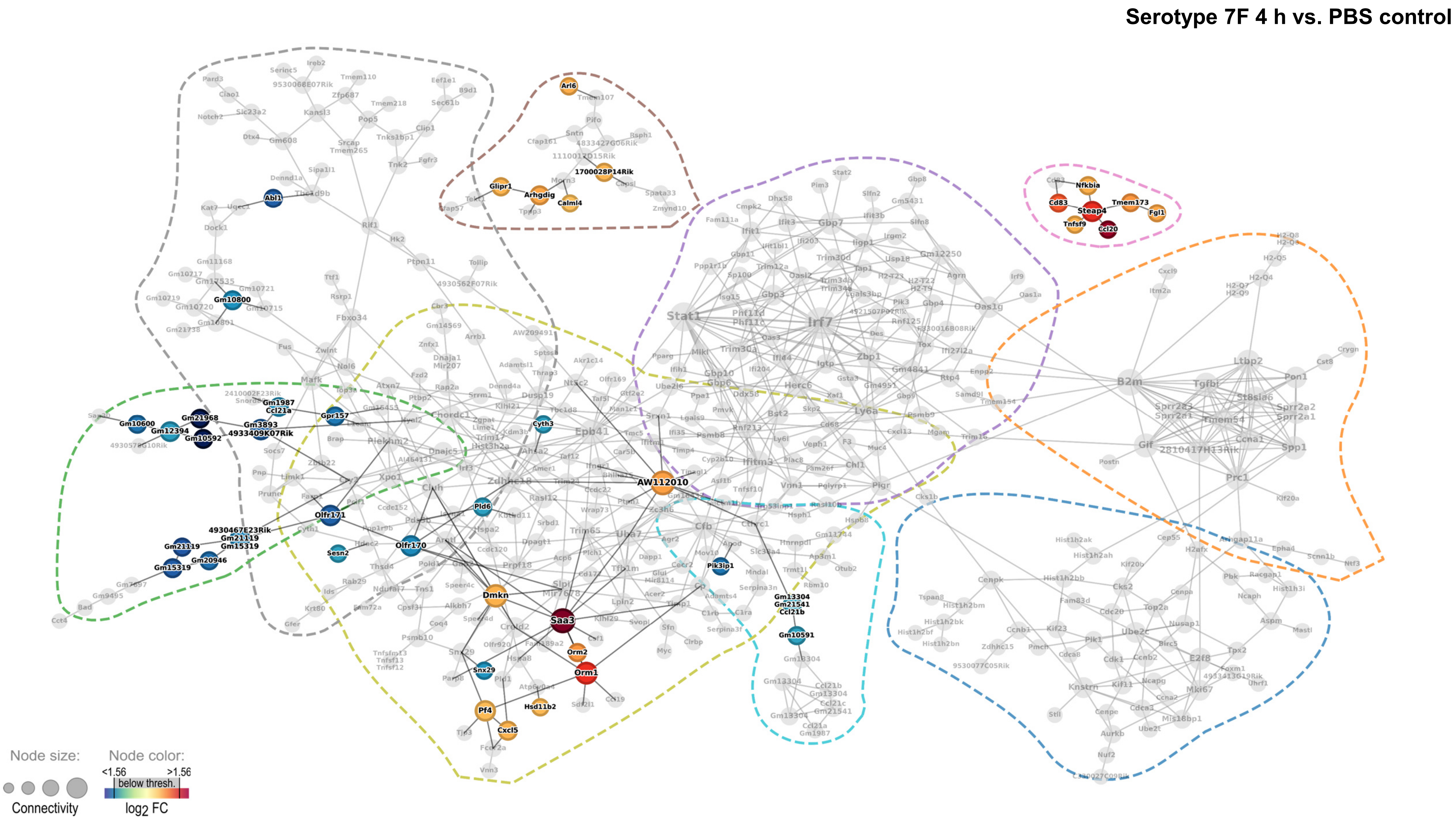

Serotype 7F 4 h vs. PBS control
Node size:
Connectivity
Node color:
<1.56
>1.56
below thresh.
log2 FC
Arl6
1700028P14Rik
Glipr1
Arhgdig
Calml4
Nfkbia
Cd83
Steap4
Tmem173
Fgl1
Tnfsf9
Ccl20
Abl1
Gm10800
Gm10600
Gm12394
Gm21968
Gm10592
Gm3893
4933409K07Rik
Gm1987
Ccl21a
Gpr157
Cyth3
AW112010
Pld6
Olfr171
Olfr170
4930467E23Rik
Gm21119
Gm15319
Gm20946
Sesn2
Pik3ip1
Gm13304
Gm21541
Ccl21b
Gm10591
Dmkn
Saa3
Orm2
Orm1
Snx29
Hsd11b2
Pf4
Cxcl5

Serotype 4 4 h vs. PBS control

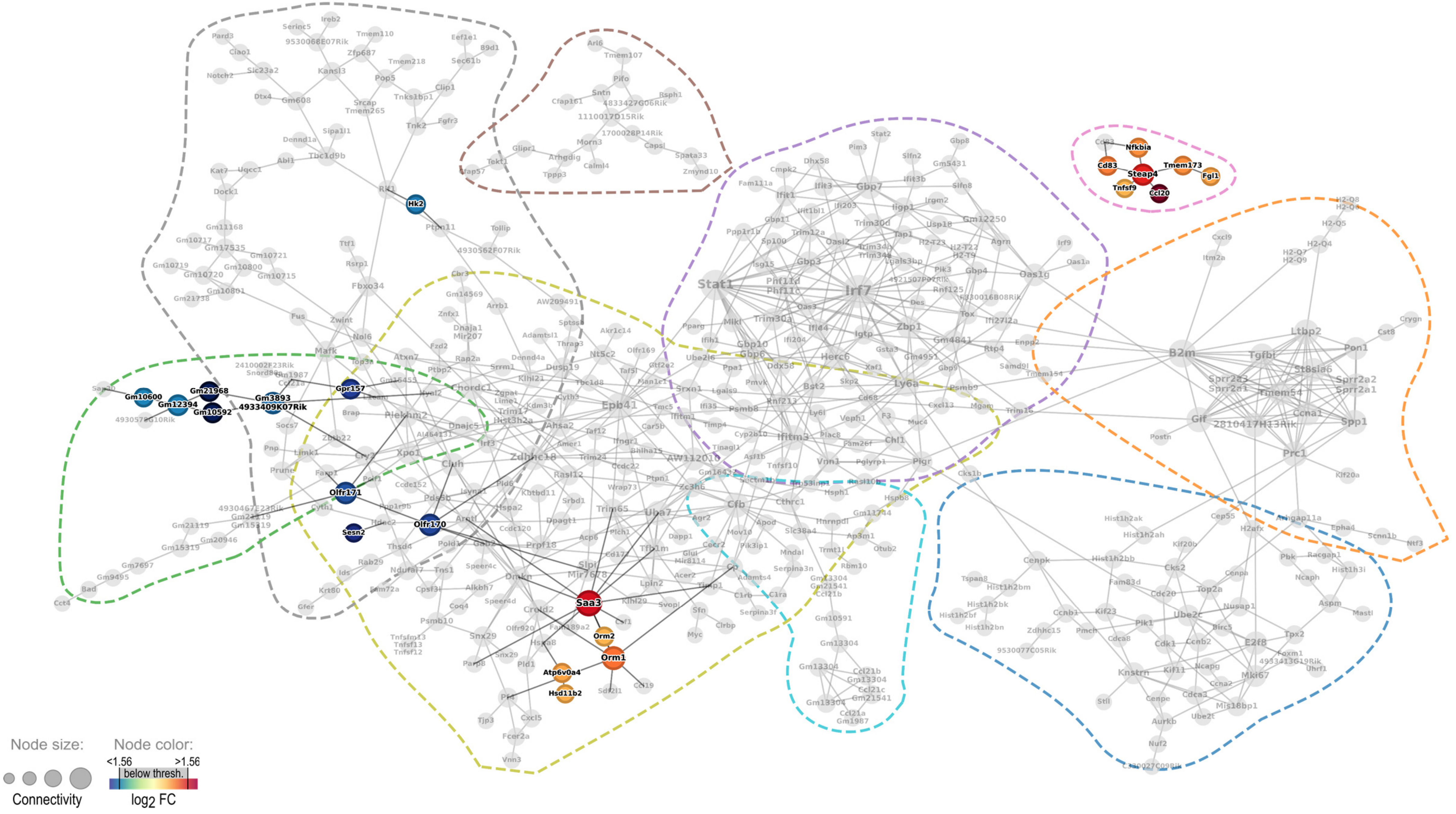

Serotype 19F 18 h vs. PBS control

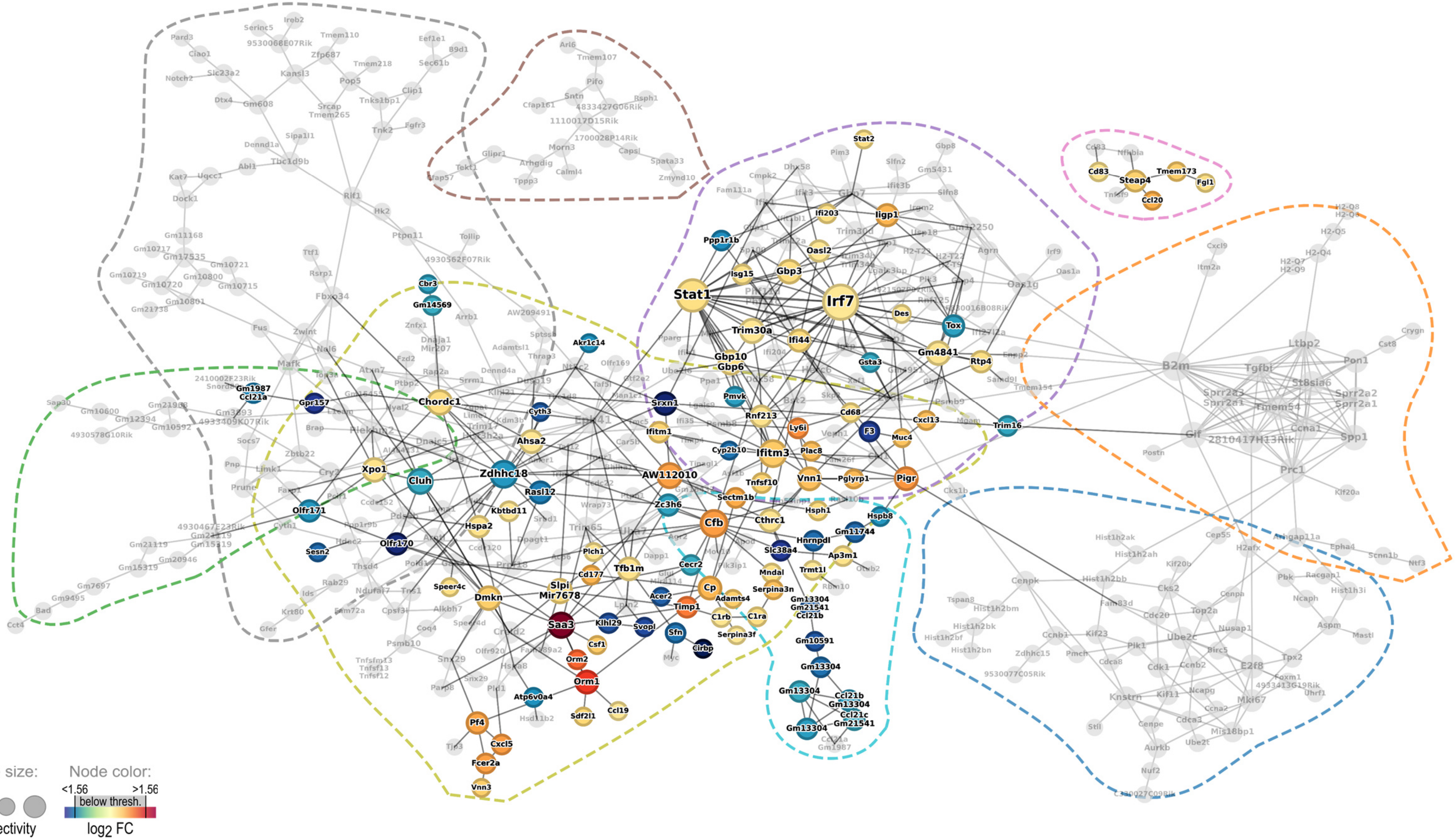

Serotype 7F 18 h vs. PBS control

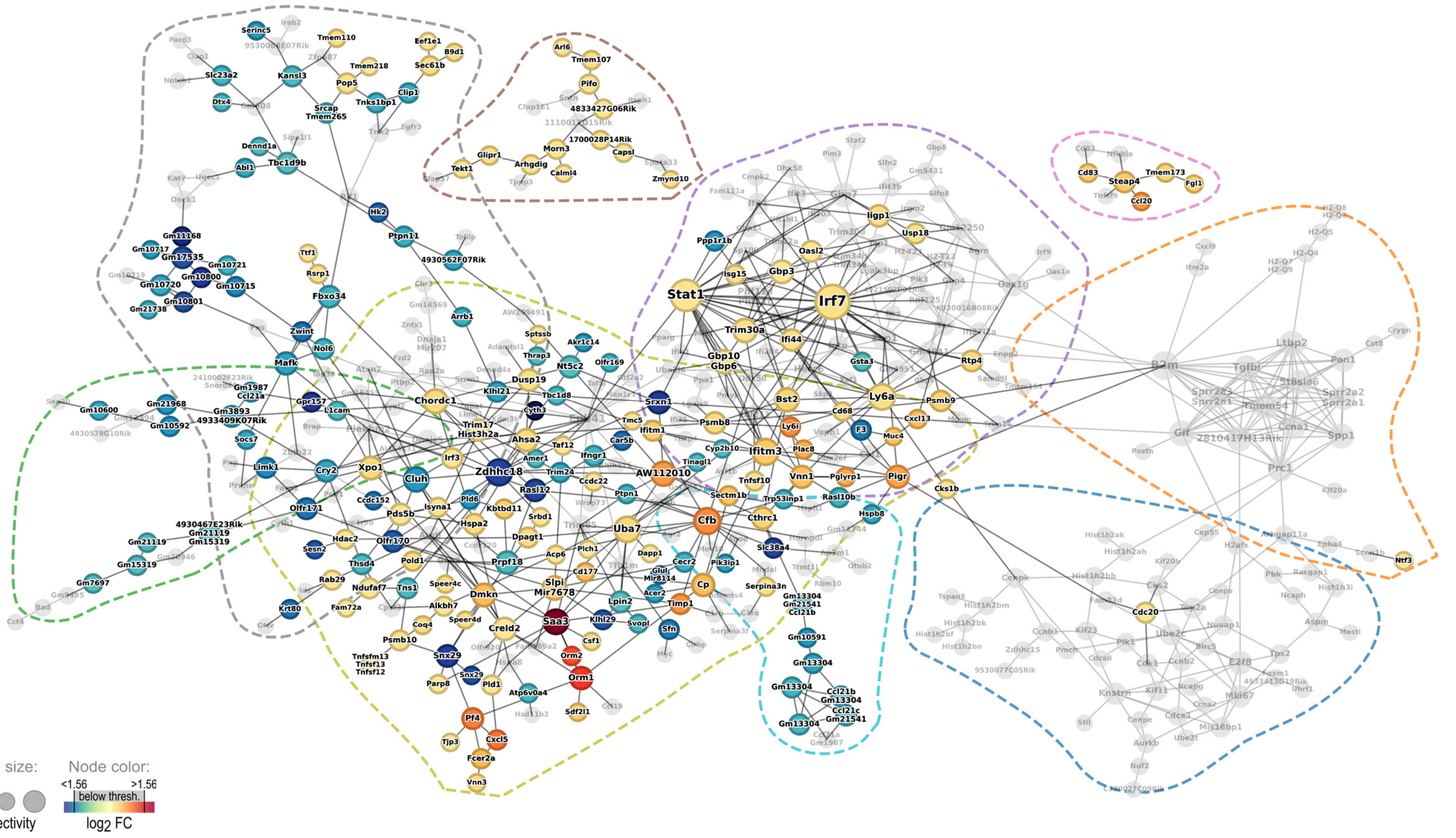

Serotype 4 18 h vs. PBS control

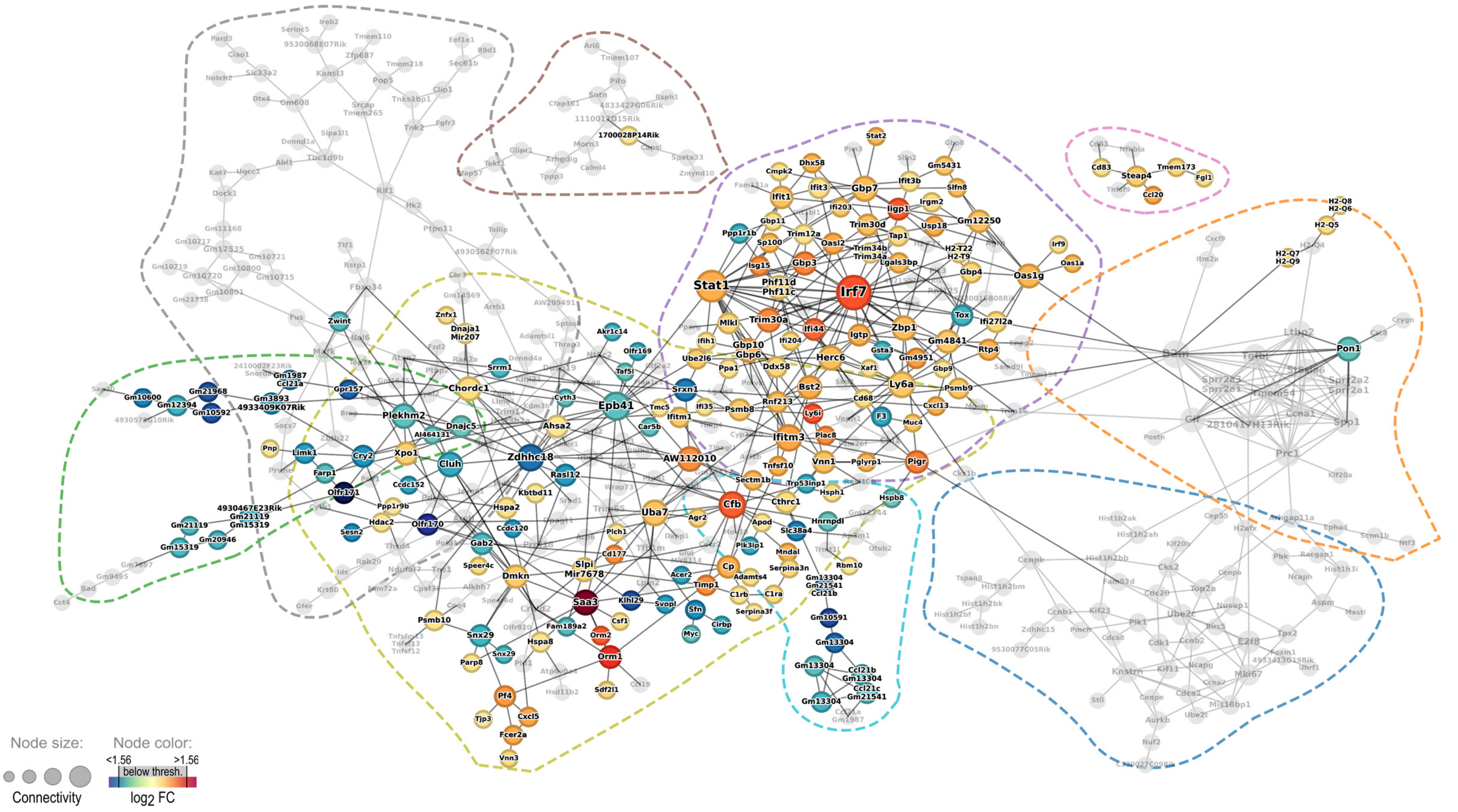

IAV day 14 vs. PBS control

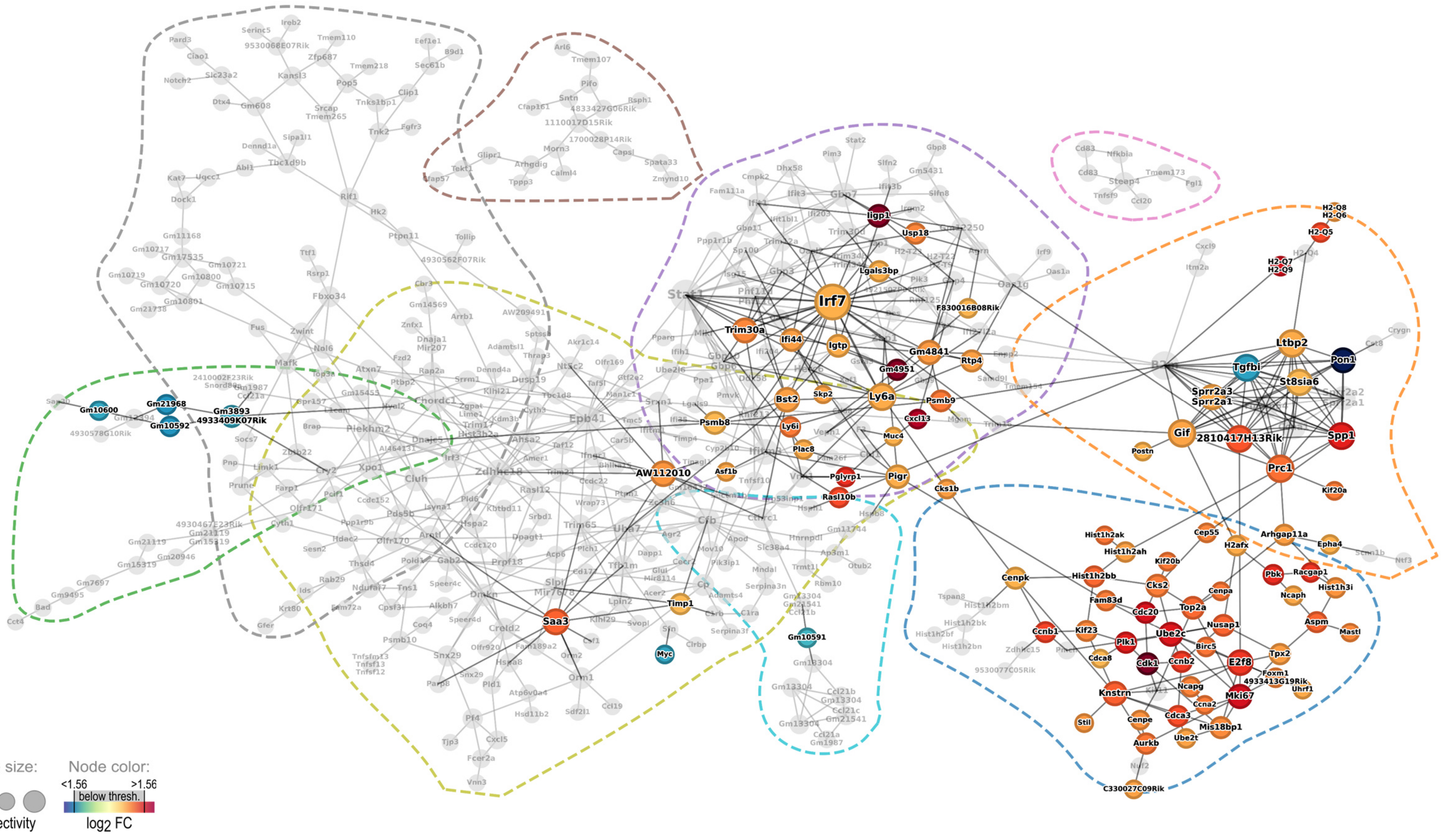

IAV day14 + Serotype 19F 4 h vs. PBS control

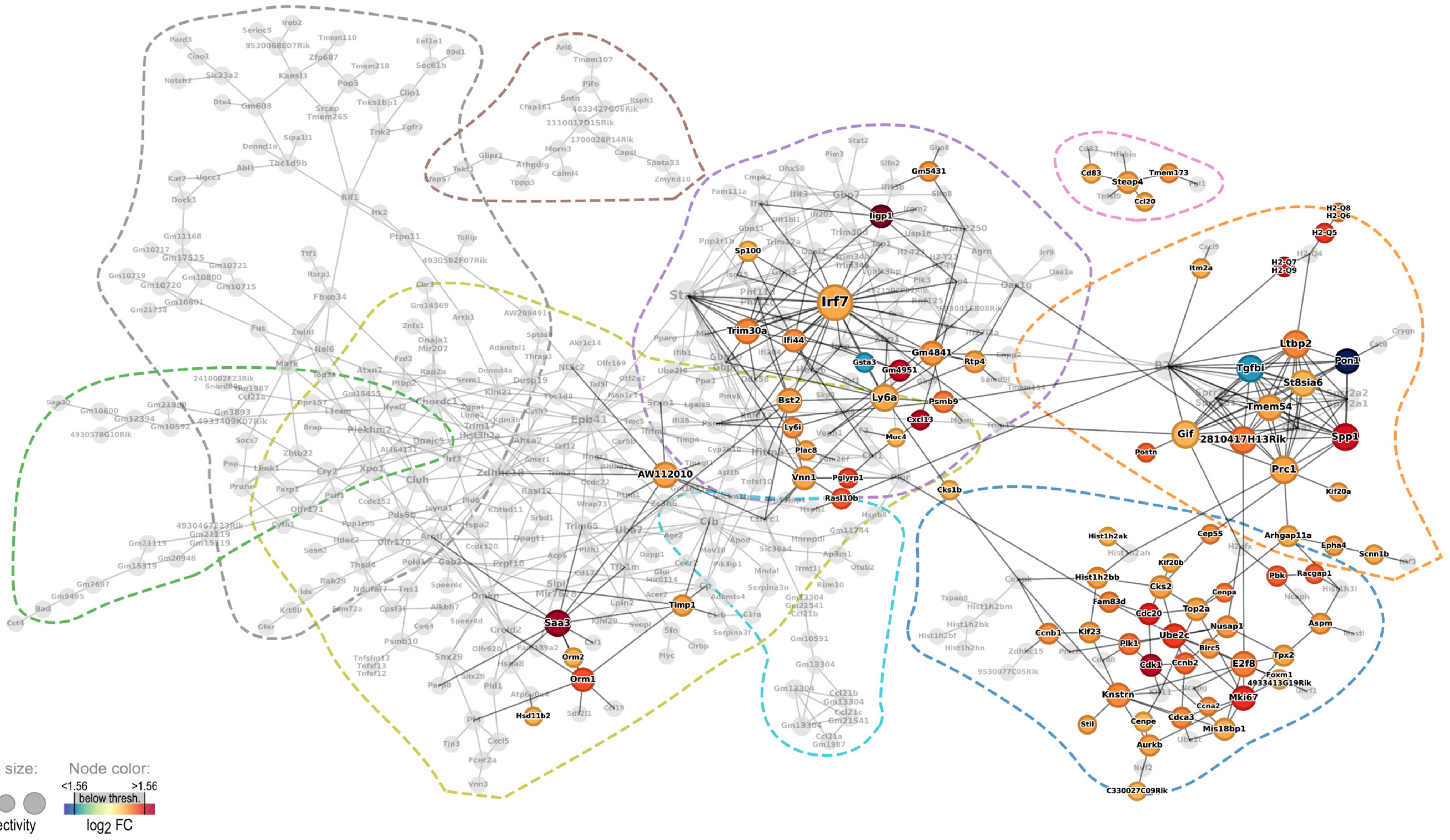

IAV day14 + Serotype 7F 4 h vs. PBS control

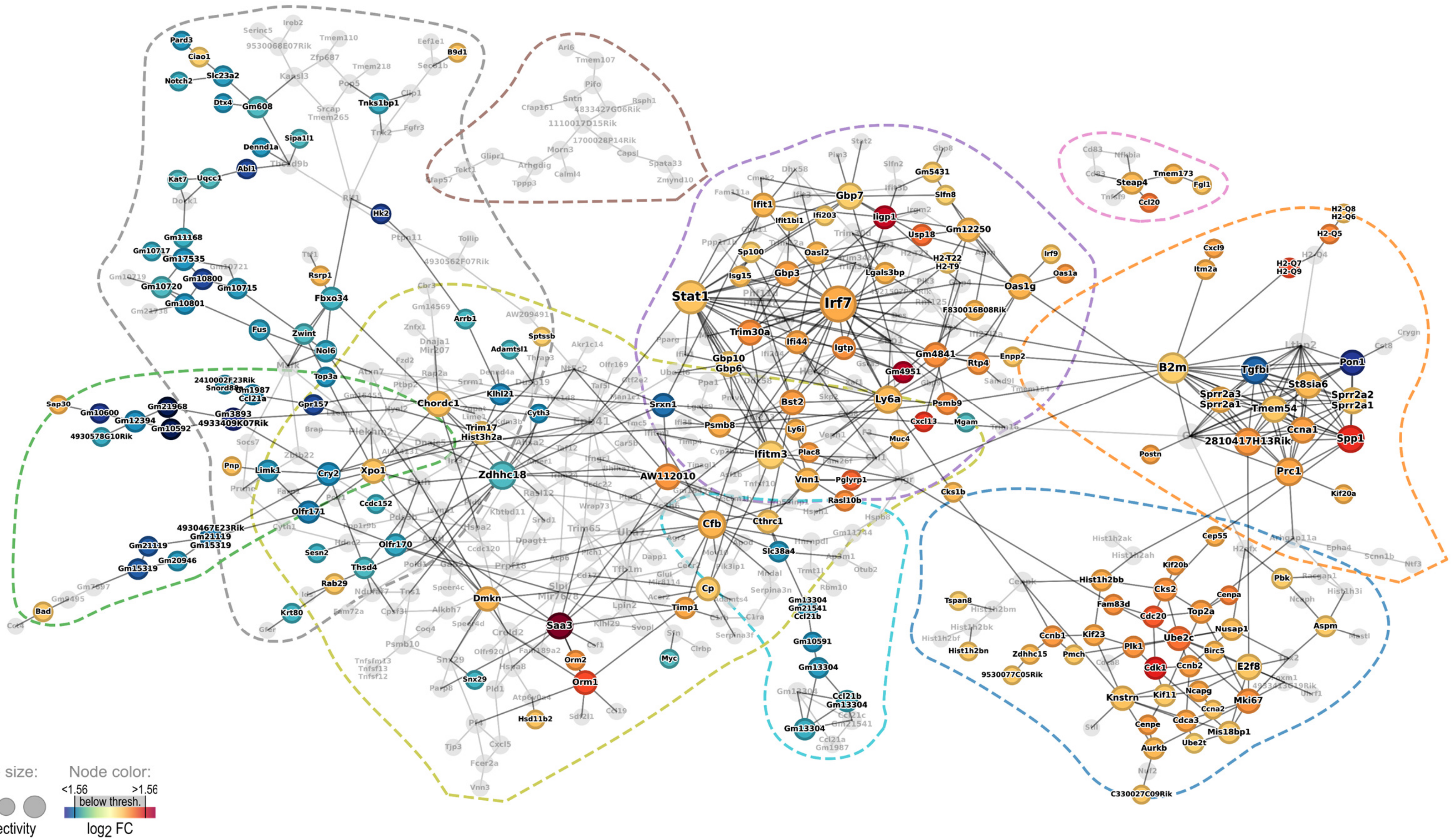

IAV day14 + Serotype 4 4 h vs. PBS control

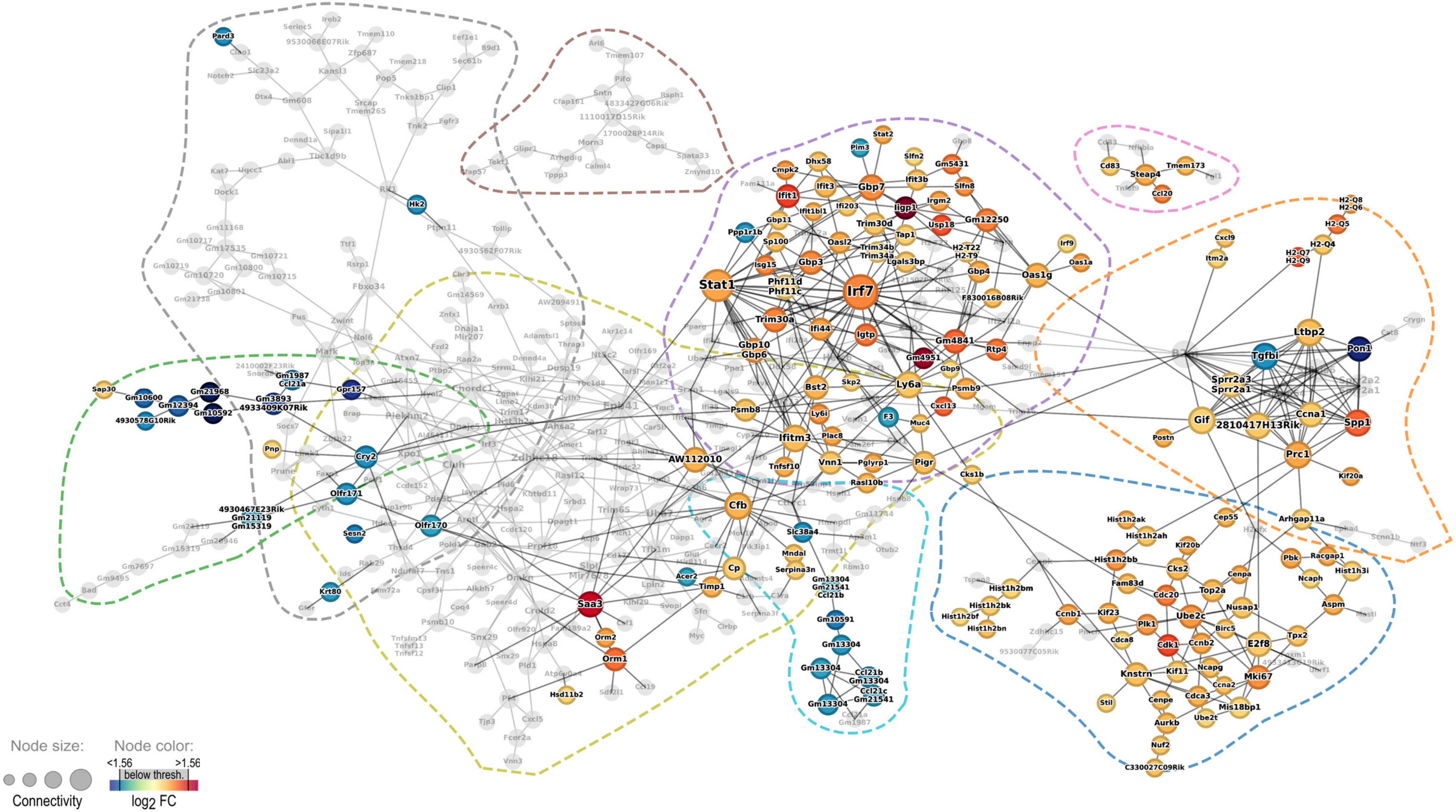

IAV day14 + Serotype 19F 18 h vs. PBS control

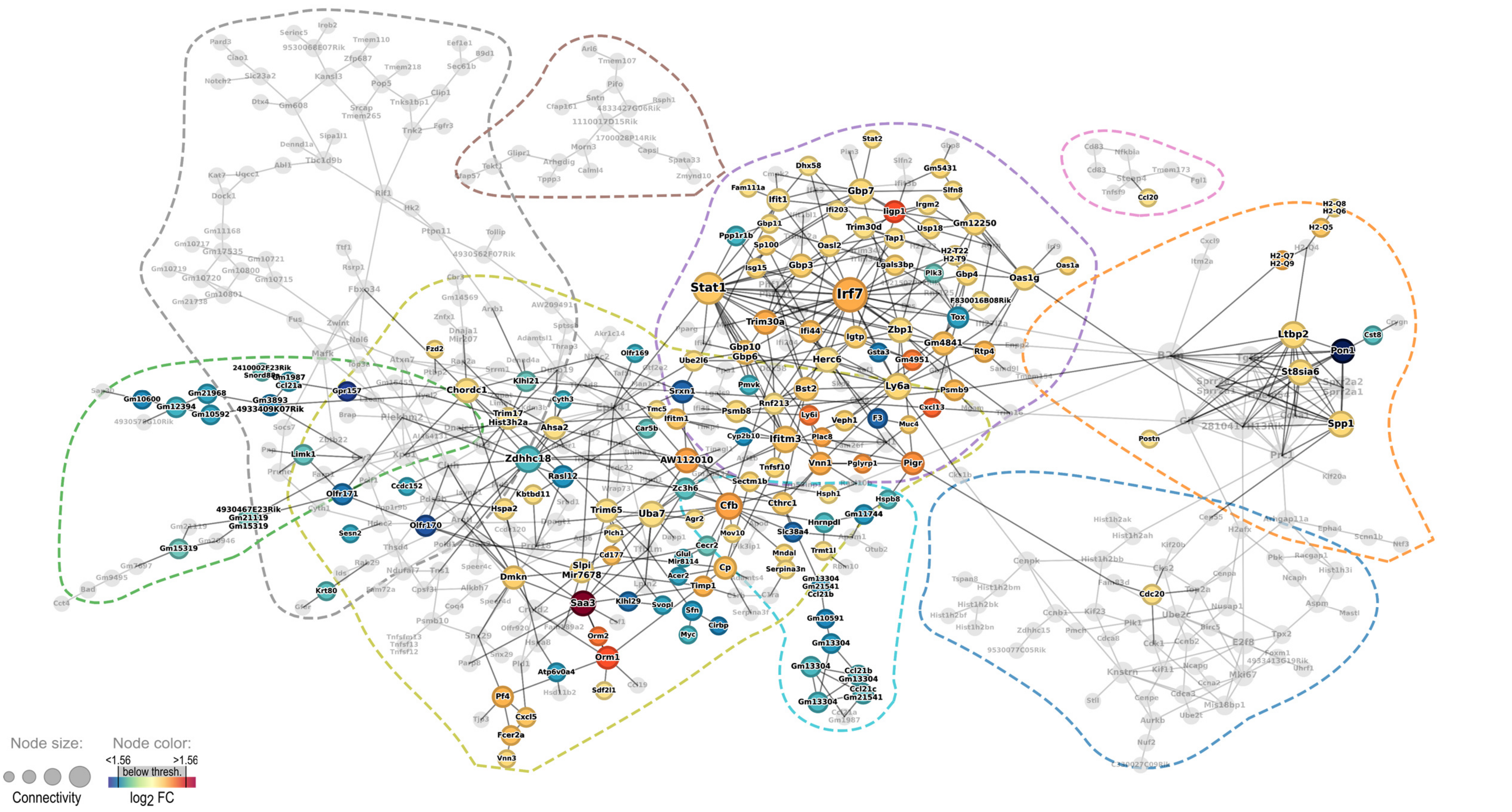

IAV day14 + Serotype 7F 18 h vs. PBS control

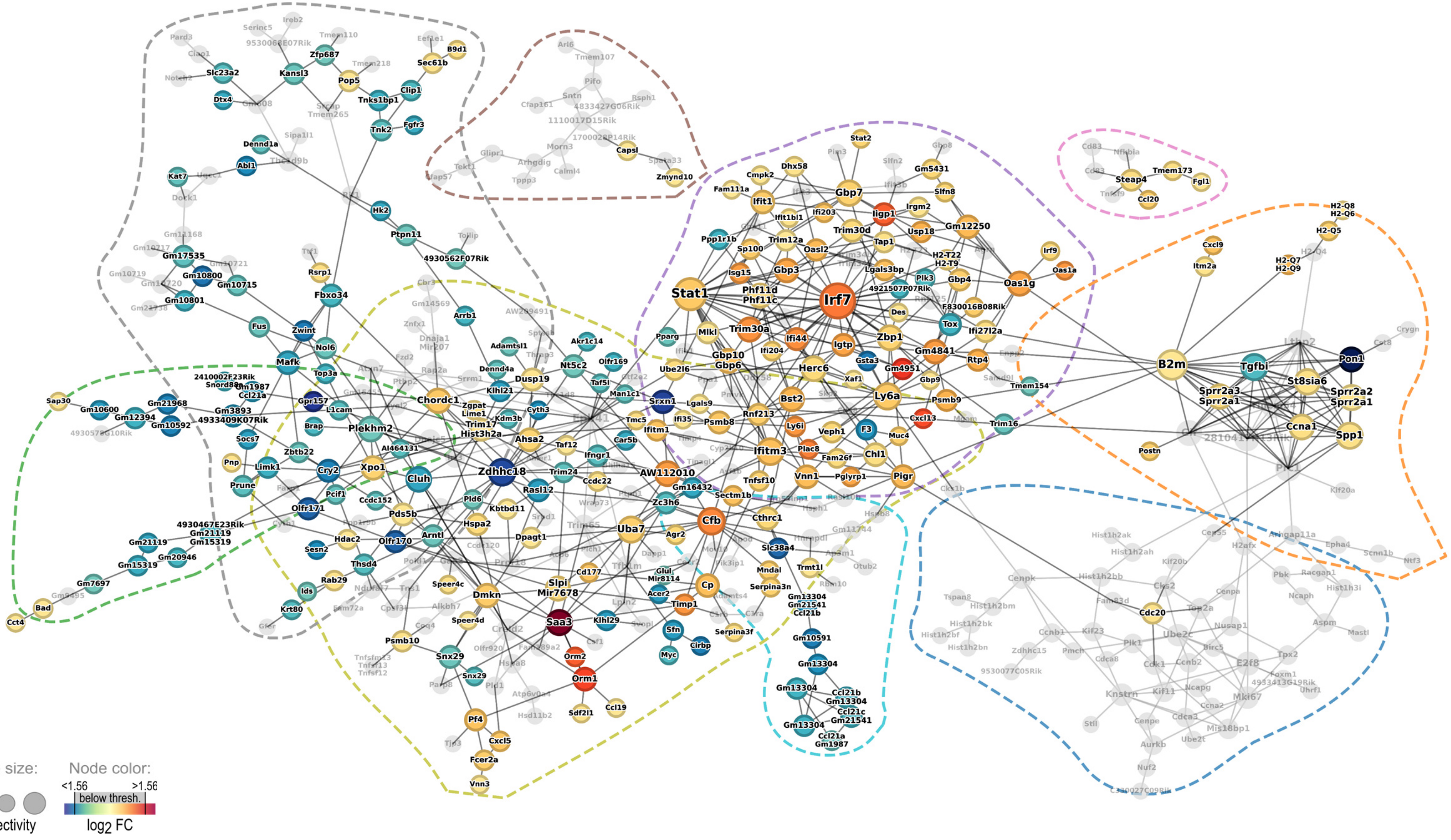

IAV day 14 + Serotype 4 18 h vs. PBS control

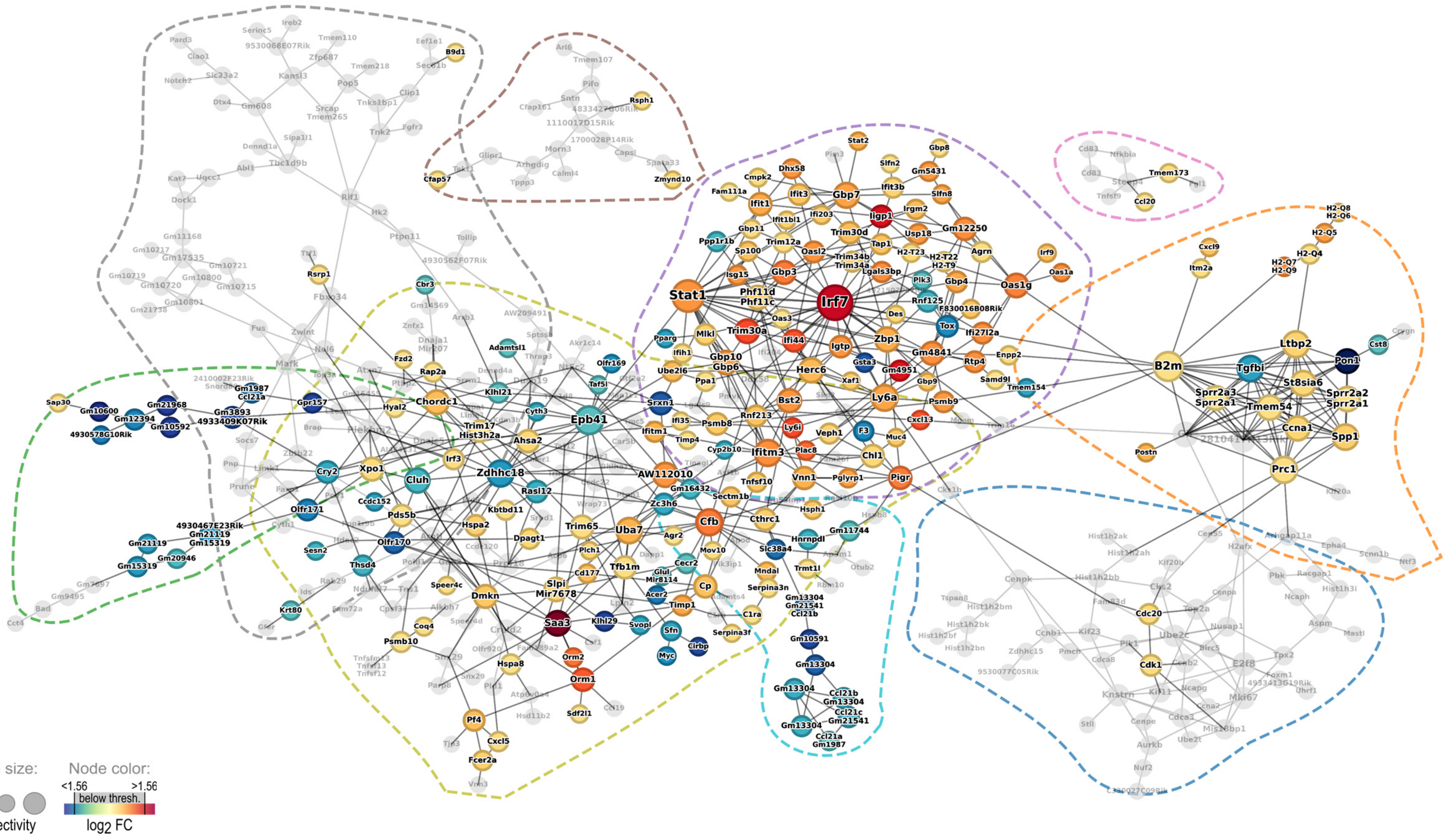

Supplement: Supplementary file 8 — Additional file 8: Differential AECII ARACNE gene co-expression partial networks. [file 12964_2025_2284_MOESM8_ESM.pdf]
