# Supplementary material for: Epigenetic changes and serotype-specific responses of alveolar type II epithelial cells to Streptococcus pneumoniae in resolving influenza A virus infection
Source: Cell Commun Signal. 2025 Jun 12;23:278. doi: 10.1186/s12964-025-02284-y (PMC12164077; doi:10.1186/s12964-025-02284-y)

Additional file 9: Clustering coefficients of ARACNE network nodes. Nodes are color-coded according to their clustering coefficient. Node size indicates node connectivity. Colored dashed lines indicate outlines of network modules. Nodes are labeled with gene symbols.

Clustering coefficients of ARACNE network nodes

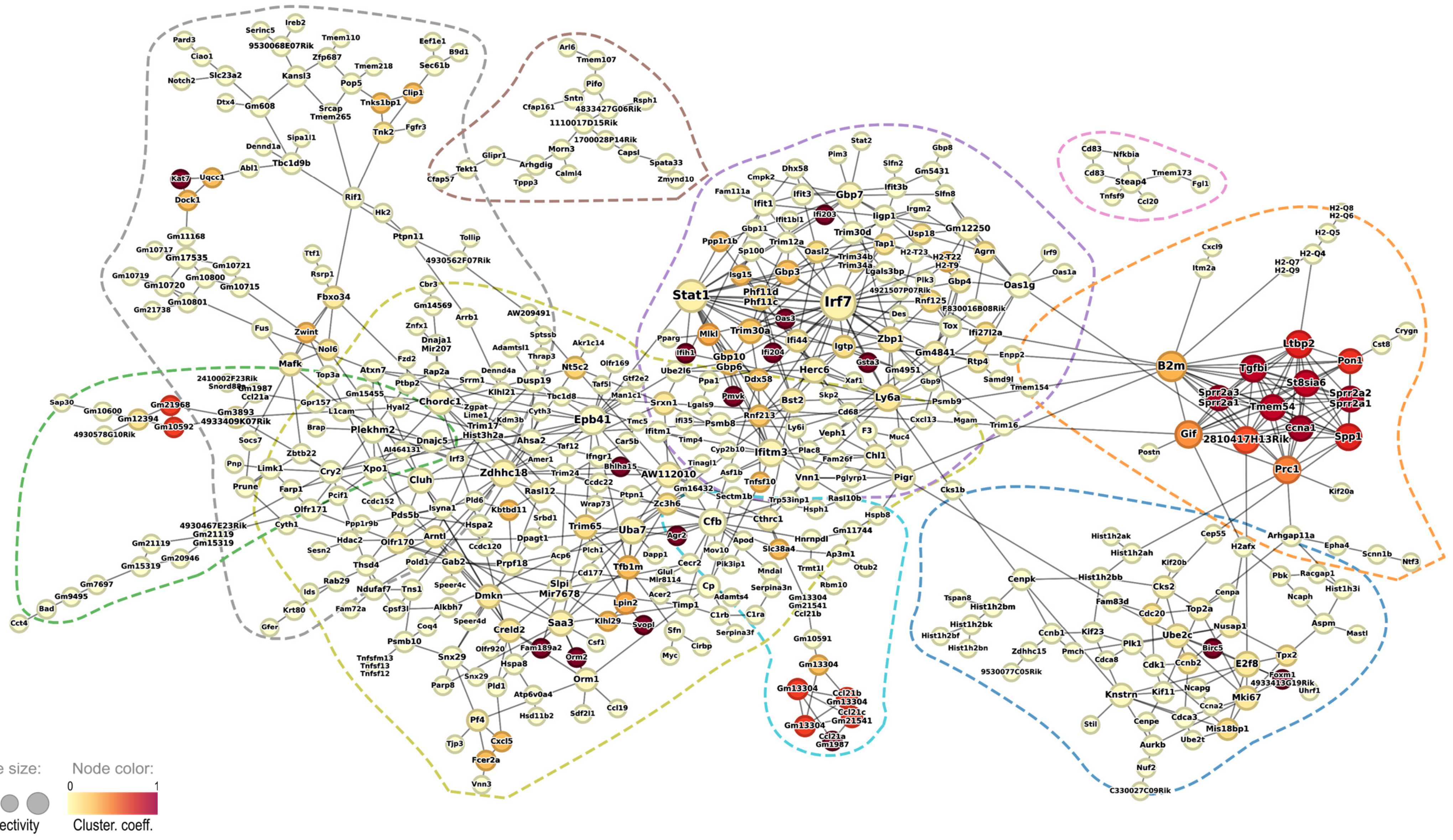

Supplement: Supplementary file 9 — Additional file 9: Clustering coefficients of ARACNE network nodes. [file 12964_2025_2284_MOESM9_ESM.pdf]
