# Supplementary material for: Epigenetic changes and serotype-specific responses of alveolar type II epithelial cells to Streptococcus pneumoniae in resolving influenza A virus infection
Source: Cell Commun Signal. 2025 Jun 12;23:278. doi: 10.1186/s12964-025-02284-y (PMC12164077; doi:10.1186/s12964-025-02284-y)
Supplement: Supplementary file 13 — Additional file 13: Additional methods: Viral burden quantification by quantitative real-time RT-PCR. [file 12964_2025_2284_MOESM13_ESM.pdf]

Supplementary Material

Viral burden quantification by quantitative real-time RT-PCR

Lungs were perfused using 10mL ice-cold PBS, single lobes were excised, rinsed with PBS and transferred into 4mL RNA*later*^{TM} stabilization reagent (Invitrogen^{TM}) followed by overnight incubation at 4°C and storage at -20°C until further use. Stabilized lobes were homogenized in 7mL buffer RLT Plus (Qiagen) containing 1% (v/v) β-Mercaptoethanol. Debris was removed by centrifugation and RNA was isolated using the RNease Plus kit (Qiagen) according to the manufacturer's instructions. Quantitative real-time RT-PCR was performed using the SensiFAST^{TM} SYBR No-ROX One-Step kit (Bioline) and a LightCycler 480 II instrument (Roche). For each sample the same RNA concentration (130ng) was used in conjunction with a plasmid standard containing defined numbers of the IAV *NP* gene as previously described [1]. Primer sequences *Influenza nucleoprotein* (*NP*): *NP*-5' gaggggtgagaatggacgaaaaac; *NP-3'* caggcaggcaggcaggactt.

Reference

1. Stegemann-Koniszewski S, Jeron A, Gereke M, Geffers R, Kröger A, Gunzer M, et al. Alveolar Type II Epithelial Cells Contribute to the Anti-Influenza A Virus Response in the Lung by Integrating Pathogen- and Microenvironment-Derived Signals. mBio. 2016 May 3;7(3):e00276-16.
