# Supplementary material for: Epigenetic changes and serotype-specific responses of alveolar type II epithelial cells to Streptococcus pneumoniae in resolving influenza A virus infection
Source: Cell Commun Signal. 2025 Jun 12;23:278. doi: 10.1186/s12964-025-02284-y (PMC12164077; doi:10.1186/s12964-025-02284-y)

Additional file 10: Clustered node neighbour expression heatmaps of interferon-hub genes *Irf7* and *Stat1*. Z-scores of averaged normalized log_2 signal intensities of node neighbours of *Irf7* and *Stat1* in the ARACNE network were hierarchically clustered and color coded. (+)/(-) behind genes symbols indicates positive/negative correlation of stated gene with *Irf7* or *Stat1*, respectively.

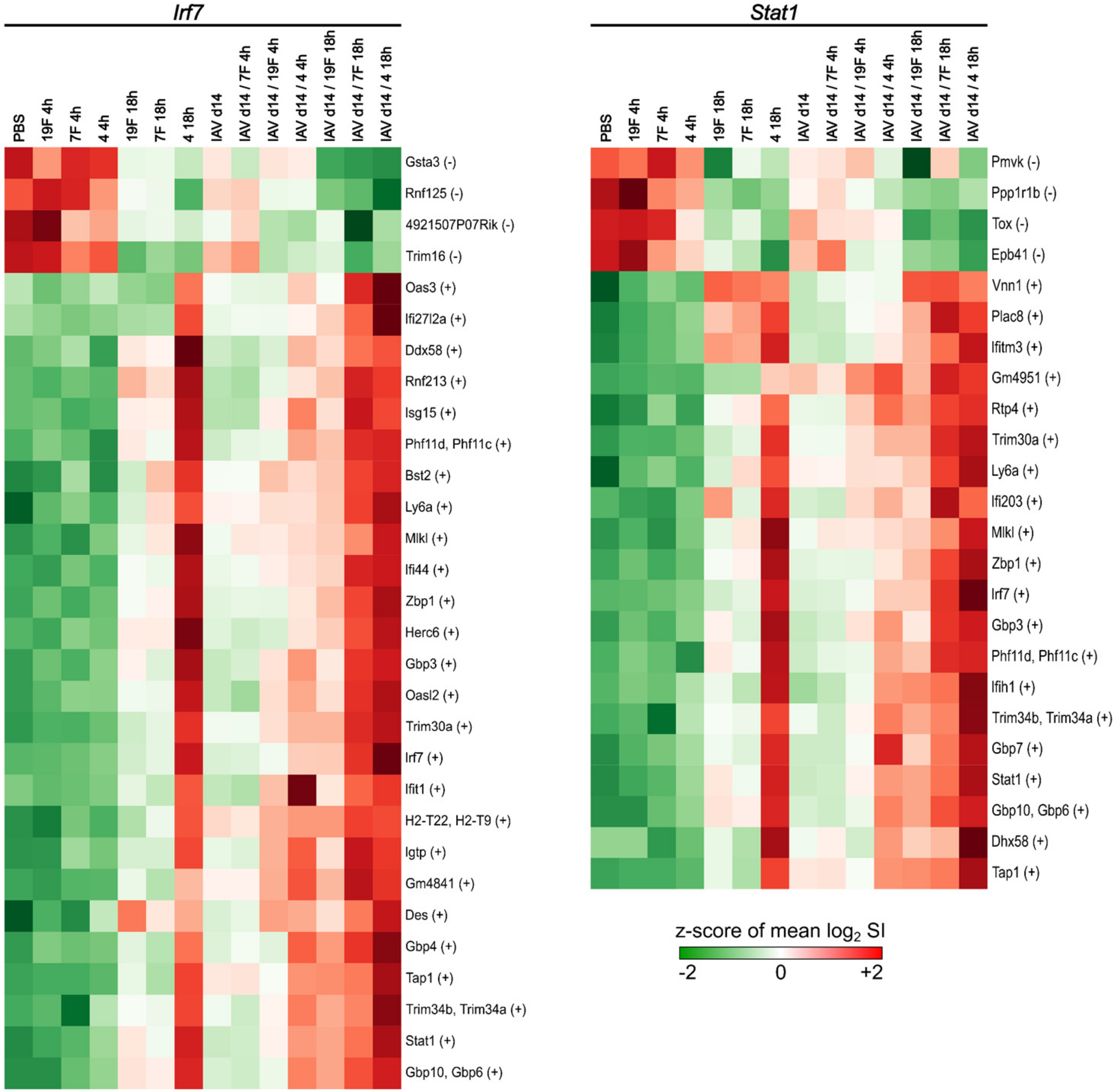

Supplement: Supplementary file 10 — Additional file 10: Clustered node neighbor expression heatmaps of interferon-hub genes Irf7 and Stat1. [file 12964_2025_2284_MOESM10_ESM.pdf]
